# Engineering Photoluminescence of Lanthanide Doped Yttrium-MOF-76 for Volatile Organic Compound Sensing

**DOI:** 10.3390/polym17091135

**Published:** 2025-04-22

**Authors:** Oswaldo Rosas Rivas, Mariana Hamer, Héctor A. Baldoni, Maya Boone, Rik Van Deun, Germán E. Gomez

**Affiliations:** 1 Área de Química General e Inorgánica “Dr. G. F. Puelles”, Facultad de Química, Bioquímica y Farmacia, Universidad Nacional de San Luis, Ejército de los Andes 950, San Luis 5700, Argentina; ojrrt55@gmail.com (O.R.R.); hbaldoni@unsl.edu.ar (H.A.B.); 2 Instituto de Investigaciones en Tecnología Química (INTEQUI), Almirante Brown 1455, San Luis 5700, Argentina; 3 Área Química, Instituto de Ciencias, Universidad Nacional de General Sarmiento, CONICET. J. M. Gutiérrez 1150, Buenos Aires 1613, Argentina; mhamer@campus.ungs.edu.ar; 4 Instituto Multidisciplinario de Investigaciones Biológicas de San Luis (IMIBIO-SL), CONICET-UNSL, Av. Ejército de los Andes 950, San Luis 5700, Argentina; 5 L3—Luminescent Lanthanide Lab, Department of Chemistry, Ghent University, Krijgslaan 281, Building S3, 9000 Gent, Belgium; maya.boone@ugent.be (M.B.); rik.vandeun@ugent.be (R.V.D.)

**Keywords:** MOF, lanthanides, luminescence, sensing, VOCs

## Abstract

A set of three-dimensional metal-organic frameworks, named MOF-76, belonging to the tetragonal P4_3_22 space group, based on [Y(BTC)(H_2_O)](DMF)_1.1_ (1,3,5-benzenetricarboxylate) doped with Eu^3+^, Tb^3+^, and Eu^3+^/Tb^3+^ were obtained under solvothermal conditions and fully characterized by powder X-ray diffraction, thermal, and vibrational analyses. In addition, upon UV light excitation (280 nm), all the powdered samples exhibited fine 4f-4f transitions, of which the ^5^D_0_ → ^7^F_2_ (Eu^3+^) and ^5^D_4_ → ^7^F_5_ (Tb^3+^) were the most intense ones. All samples were photophysically analyzed by determining the luminescence lifetimes, and their emission colors were quantified by calculating their chromaticities and color purities. Moreover, the intrinsic quantum yield, radiative, and non-radiative constants were calculated and compared to establish a structure–property relationship. Specifically, the Eu/Tb co-doped sample was employed to monitor its hypersensitive emissions in the presence of small volatile organic compounds (VOCs), showing quenching or enhancement of emission in protic and non-protic solvents. Furthermore, DFT calculations were carried out to understand the energy transfer processes between the sensor and the respective analytes. These results are promising for the development of solid-state lighting devices and colorimetric chemical sensors for specific compounds.

## 1. Introduction

Metal–organic frameworks (MOFs) are a type of coordination polymer that have attracted significant attention due to their diverse applications, including ion exchange [1,2], gas separation [3], heterogeneous catalysis [4], optoelectronics [5], optomagnetism [6], controlled drug release [7], and antimicrobial composites for human health [8]. These materials stand out for their high porosity, large surface area, and structural flexibility, enabling the design of micro- and nanomaterials with tailored properties derived from metal ions, organic linkers, and their synergistic interactions [9,10,11].

Among MOFs, lanthanide-based metal–organic frameworks (Ln-MOFs) are particularly valued for photonics and magnetism, which are attributed to the unique 4f-4f electronic configuration. The 4f-4f transitions result in sharp spectral lines, varied lifetimes, and high quantum yields, making Ln-MOFs ideal for applications such as phosphors, lasers, optical amplifiers, solid-state lighting, full-color displays, and backlighting systems [5,12,13,14]. Moreover, there are numerous examples of Ln-MOFs employed as models for water remediation by photocatalysis [15].

Furthermore, mixed-lanthanide MOFs offer enhanced functionality compared to single-lanthanide counterparts, enabling tunable white light emission and efficient temperature sensing, thereby broadening their potential applications in fields requiring precise optical responses [3,7,16].

Within Ln-MOFs, MOF-76 stands out for its remarkable structural and optical properties. This structure, based on 1,3,5-benzenetricarboxylate (BTC^3−^) ligands coordinated to lanthanide centers, crystallizes in the tetragonal P4_3_22 (#95) space group and presents well-defined porous 1D channel structure cavities [17,18,19]. Additionally, Eu-MOF-76 and Tb-MOF-76 exhibit notable 4f-4f emissions, such as red emission at 610 nm (Eu^3^⁺) and green emission at 540 nm (Tb^3^⁺), which are further amplified by the “antenna effect”, where organic ligands efficiently transfer energy to lanthanide ions, enhancing their photoluminescence intensity [18]. These attributes make MOF-76 a promising candidate for applications in chemical and thermal sensors, photocatalysis, and light-emitting devices [20].

One of the most pressing applications of MOF materials lies in the detection and recognition of volatile organic compounds (VOCs), which are critical for environmental monitoring, industrial safety, and public health [21]. VOCs are emitted from a plethora of industrial processes, household products, and natural sources, contributing to air pollution and posing serious health risks, including respiratory issues, neurological disorders, and even cancer with prolonged exposure [22]. Additionally, VOCs play a crucial role in atmospheric chemistry, participating in reactions that lead to the formation of ground-level ozone and secondary organic aerosols, which exacerbate air quality problems [23].

Despite the urgent need for effective VOC sensing technologies, existing detection methods face significant challenges. Traditional techniques such as gas chromatography and mass spectrometry provide high sensitivity and selectivity; however, they are often expensive, time-consuming, and require specialized instrumentation, limiting their practicality for real-time or on-site monitoring [24,25,26,27]. Moreover, conventional solid-state sensors, including metal oxide and electrochemical sensors, frequently suffer from poor selectivity, slow response and recovery times, and sensitivity to environmental factors such as humidity and temperature fluctuations, leading to inconsistent performance [28]. These limitations highlight the need for alternative sensing platforms that combine high sensitivity, selectivity, rapid response, and recyclability.

MOFs have demonstrated exceptional potential as VOC sensors due to their high porosity, selective adsorption capabilities, chemical and thermal stabilities, and tunable luminescent properties [29,30]. The ability of luminescent MOFs to interact with VOCs through host–guest interactions enables highly sensitive and selective detection, as analyte adsorption can modulate photoluminescence properties through enhancement or quenching, wavelength shifts, or lifetime variations, providing a sensitive and selective detection mechanism [31]. However, achieving precise control over these luminescent responses and understanding the fundamental mechanisms governing them remain ongoing challenges.

In this study, we explore a novel application of MOF-76 doped with europium and terbium for solvent-specific sensing devices. Powder X-ray diffraction, thermogravimetric analysis, differential scanning calorimetry, and infrared spectroscopy were employed to assess the material’s structural, vibrational, and thermal properties. An in-depth photophysical characterization was carried out by recording excitation and emission spectra, calculating the lifetime values, and europium’s intrinsic quantum yields. Also, the lanthanide energy transfer and color quantification were estimated. Finally, the luminescent response of the Eu/Tb co-doped MOF-76 in various solvents was evaluated, demonstrating its potential for developing advanced chemical sensors capable of detecting VOCs.

## 2. Materials and Methods

### 2.1. Synthesis

All reagents and solvents were used as received from Sigma-Aldrich (Burlington, MA, USA) without further purification: EuCl_3_∙6H_2_O, TbCl_3_∙6H_2_O, YCl_3_.6H_2_O, N,N′-dimethylformamide (DMF), and 1,3,5-benzenetricarboxylic acid (H_3_BTC, H_6_C_9_O_6_). The crystalline materials were obtained as crystalline solids under solvothermal conditions using 43 Parr reactors. Compounds with formula [Ln(C_9_H_3_O_4_)(H_2_O)]∙(DMF)_1.1_ (further labeled as Ln-BTC) were obtained following previously reported procedures with slight modifications [32,33].

#### 2.1.1. Y-BTC

H_3_BTC (0.60 mmol, 0.1208 g) and YCl_3_·6H_2_O (0.50 mmol, 0.1517 g) were dissolved in DMF (9 mL) and H_2_O (3 mL) at room temperature. The mixture was stirred for 30 min, then transferred to a Teflon-lined Parr reactor and heated at 80 °C for 72 h. After that, the product was filtered and washed with 10 mL of water and 10 mL of DMF. Finally, the crystalline product was dried at room temperature for 48 h.

#### 2.1.2. Ln@Y-BTC

For the synthesis of lanthanide-doped compounds, a similar procedure to Y-BTC was followed, except for the addition of 5% of EuCl_3_·6H_2_O (0.0201 g) (**Eu@Y-BTC**) or TbCl_3_·6H_2_O (0.0204 g) (**Tb@Y-BTC**). For co-doped samples, **Eu_2.5_Tb_2.5_@Y-BTC** was prepared using the same procedure explained before, but including EuCl_3_·6H_2_O in a 2.5% (0.0101 g) and 2.5% TbCl_3_·6H_2_O (0.0104 g) amount. Also, **Eu_1.25_Tb_3.75_@Y-BTC** was prepared by incorporating 1.25% EuCl_3_·6H_2_O (0.050 g) and 2.5% TbCl_3_·6H_2_O (0.0154 g). The incorporation of Eu and Tb into the mixed MOFs was confirmed by ICP analysis. As shown in Table S4, the measured percentages of the dopant elements are slightly lower than the nominal amounts used in the synthesis. However, the Eu:Tb ratio remains consistent with the intended doping ratio, indicating a uniform incorporation of both lanthanides into the frameworks.

### 2.2. Characterization

The powder X-ray diffraction (PXRD) plots were recorded with a Rigaku–Ultima IV type II diffractometer. A scanning step of 0.05° into the 5–50 2-theta Bragg angles range with an exposure time of 5 s per step was used to obtain the best counting statistics. Fourier transform infrared (FTIR) spectra were recorded with a Nicolet Protégé 460 spectrometer in the 4000–400 cm^−1^ range with 64 scans and a spectral resolution of 4 cm^−1^ by the KBr pellet technique. Thermogravimetric analysis (TGA) was performed using a Shimadzu TGA-51 (Shimadzu Corp., Kyoto, Japan) apparatus under flowing air at a flow rate of 50 mL∙min^−1^ and a heating rate of 10 °C.min^−1^. Differential thermal analysis (DTA) was performed with a DSC-50 under air flow at a rate of 50 mL∙min^−1^ and a heating rate of 10 °C∙min^−1^.

### 2.3. Photophysical Characterization and Sensing Assays

Solid-State Luminescence Measurements: The steady-state and time-resolved luminescence measurements were performed using an Edinburgh Instruments FLSP920 spectrometer (Edinburgh Instruments Ltd, Livingston, UK) setup, using a 450 W xenon lamp as the steady-state excitation source and a 60 W pulsed xenon lamp as the time-resolved excitation source (operating at a pulse frequency of 100 Hz). The emission was detected by a Hamamatsu R928P PMT photomultiplier tube (Hamamatsu Co., Shizuoka, Japan). Excitation spectra were corrected for the xenon lamp emission profile, whereas emission spectra were corrected for the detector response curve. All measurements were carried out at a step size of 0.1 nm. Commission Internationale de l’Eclairage (CIE) (x,y) color coordinates were calculated using the Matlab (Version R2020b) program.

#### 2.3.1. Chemical Sensor Studies

The sensing activity of **Eu_1.25_Tb_3.75_@Y-BTC** was investigated by monitoring the emission spectra at 613 nm when exciting the samples at 280 nm. A quartz cuvette with a 1 cm optical path length was employed. The **VOC@Eu_1.25_Tb_3.75_@Y-BTC** suspensions were prepared by introducing 0.2 mg of powdered sample into 4 mL (0.05 mg∙mL^−1^) of each solvent [bidistilled water, methanol (Carl Roth, Karlsruhe, Germany, ≥99%), acetonitrile (Sigma Aldrich, Burlington, MA, USA, ≥99.9%) ethanol (Fischer Chemical, Pittsburgh, PA, USA, 99.9%), dimethylformamide (Sigma Aldrich, 99.8%), acetone (Acros Organics, Pittsburgh, PA, USA, pure), chloroform (Sigma Aldrich, ≥99.8%), 1,3,5-trimethylbenzene (1,3,5-TMB) (Sigma Aldrich, 98%), and toluene (Sigma Aldrich, 99.9%). The samples were previously ultrasonicated for 30 min.

#### 2.3.2. DFT Calculations

Quantum mechanical calculations were carried out using density-functional theory (DFT) and time-dependent DFT (TDDFT) with the Gaussian 16 software package [34]. Equilibrium geometries of the triplet states were optimized under tight convergence criteria using the range-separated hybrid wB97XD functional [35]. (ESI Theoretical calculations section, Listing S1–S9). This functional, which incorporates long-range corrections and empirical dispersion terms, was selected for its ability to accurately model both electronic structure and non-covalent interactions [36]. The split-valence, triple-zeta basis set augmented with diffuse and polarization functions, 6-311++G(d,p), was used to balance computational efficiency with accuracy [37]. Vibrational frequency analyses confirmed the absence of imaginary frequencies in the optimized geometries, verifying that these structures represent true local minima and ensuring the reliability of the computed results [38] (SI Theoretical calculations section, Table S1). For excited-state calculations, the Tamm–Dancoff approximation (TDA) was employed to provide a robust framework for modelling electronic excitations, particularly in systems where single excitations dominate [39]. Analyte effects were accounted for using the polarizable continuum model (PCM) in TDDFT calculations [40]. A full population analysis was conducted to quantify charge distributions using frontier molecular orbital (FMO) theory (ESI Theoretical calculations section, Figures S1–S9). The excitation assignments were obtained from the TDDFT-derived transition densities analysis [41].

## 3. Results and Discussion

### 3.1. Synthesis

**Figure 1:**
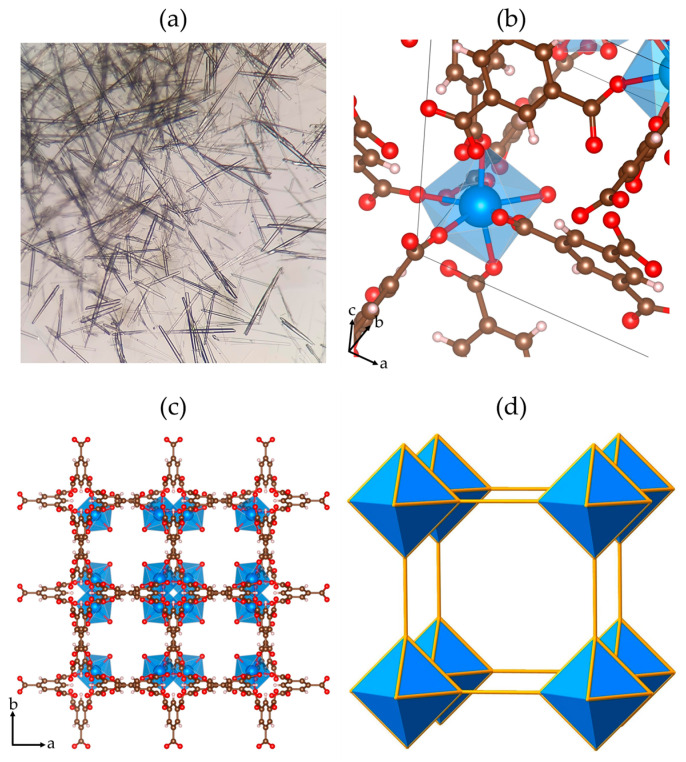
(**a**) Crystal of **Y-BTC.** (**b**) Lanthanide environment of one metallic center. (**c**) Projection on the *ab* and ac planes of **Y-BTC** (color code: brown: carbon; red: oxygen; white: hydrogen; blue: yttrium). (**d**) Topological view of the MOF-76 structure.

The described synthetic procedures led to crystalline products (Figure 1), which were fully characterized by PXRD, TGA-DSC, and FTIR techniques. According to optical microscope observations on the **Y-BTC** sample, long prismatic block crystals were obtained (Figure 1a). Additionally, a brief structural description of Ln-BTC compounds is presented. The **Ln-BTC** structure is 3D, belonging to the tetragonal P4_3_22 space group. The asymmetric unit is composed of one hepta-coordinated trivalent lanthanide ion, one BTC^3−^ linker, and one water molecule. Each lanthanide ion is surrounded by six oxygen atoms belonging to carboxylate groups and one oxygen atom from a water molecule (Figure 1b). Moreover, the metallic chain polyhedra are developed in a helical fashion along the *c* axis (Figure 1c). The chains are linked by BTC^3−^ ligands along the *a* and *b* axes, giving rise to a 3D framework. The structure contains unidimensional channels along the *c* direction, with a circular area of 36 Å^2^. The incorporation of Eu and Tb into the MOFs was confirmed by ICP-AES analysis. As shown in Table S1, the measured percentage of the dopant elements is slightly lower than the nominal amounts used in the synthesis. However, the Eu:Tb ratio remains consistent with the intended doping ratio, indicating a uniform incorporation of both lanthanides into the framework. Finally, from the topological point of view, the MOF-76 structure can be simplified into bars and dots to get the underlying net. According to Rosi et al. [17], MOF-76 belongs to the *pcu*-type net classification. This geometry consists of rods packed in a tetragonal fashion, resulting in square channels in the *c* direction, which are filled with DMF molecules. However, the rods themselves are on 4_1_ helices, but, because of the tritopic nature of the organic SBU, this results in a rather complicated overall topology.

**Figure 2:**
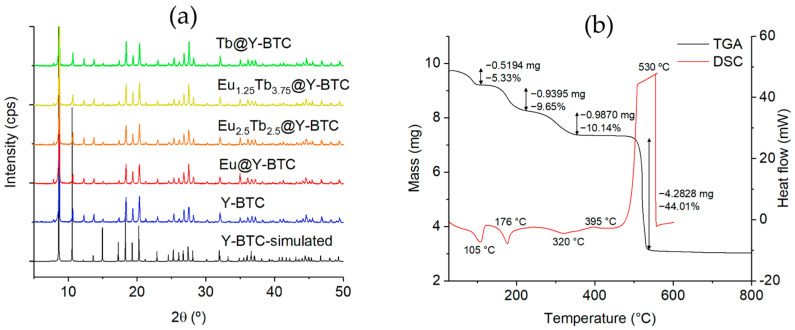
(**a**) PXRD pattern of **Ln@Y-BTC** compounds compared to the simulated one from the **Y-BTC** structure [33]. (**b**) TGA/DSC plots from the **Eu@Y-BTC** compound.

The characterization of **Ln@Y-BTC** samples by the PXRD technique revealed the isostructural nature of the reported family of compounds in comparison to the simulated pattern from the .cif file of the pristine **Y-BTC** structure (Figure 2a) [33]. The incorporation of Eu^3+^ and/or Tb^3+^ did not represent an alteration of the crystalline structure, as can be seen in the corresponding powder patterns of the sets.

Thermal analysis of the **Eu@Y-BTC** sample revealed notable stability up to 500 °C, at which point the formation of Ln₂O₃ takes place (see Figure S10). The first mass loss of 5.3% (calculated: 4.56%) in the TGA diagram corresponds to the removal of one coordinated water, accompanied by an endothermic peak in the DSC plot at 105 °C. The second and third mass loss steps, involving the removal of 1.1 DMF molecules with a mass loss of 19.7% (calculated: 20.4%), were accompanied by endothermic signals at 176 °C and 320 °C in the DSC diagram. Finally, the decomposition of the organic moieties is evidenced by a significant exothermic signal in the DSC curve at around 395 °C, and in the range of 490–550 °C, the lanthanide oxide is formed (Figure 2b). Based on these results, the final stoichiometry of **Eu@Y-BTC** was determined to be [Y_0.95_Eu_0.05_(BTC)(H_2_O)](DMF)_1.1_. Through vibrational characterization (Figure S11), it was possible to identify bands related to the asymmetric and symmetric modes of the carboxylate groups (1615, 1440, and 1380 cm^−1^) from the ligand, coordinated water (3400 cm^−1^), and guest DMF molecules (3068, 2926, and 2860 cm^−1^).

### 3.2. Photophysical Studies

**Figure 3:**
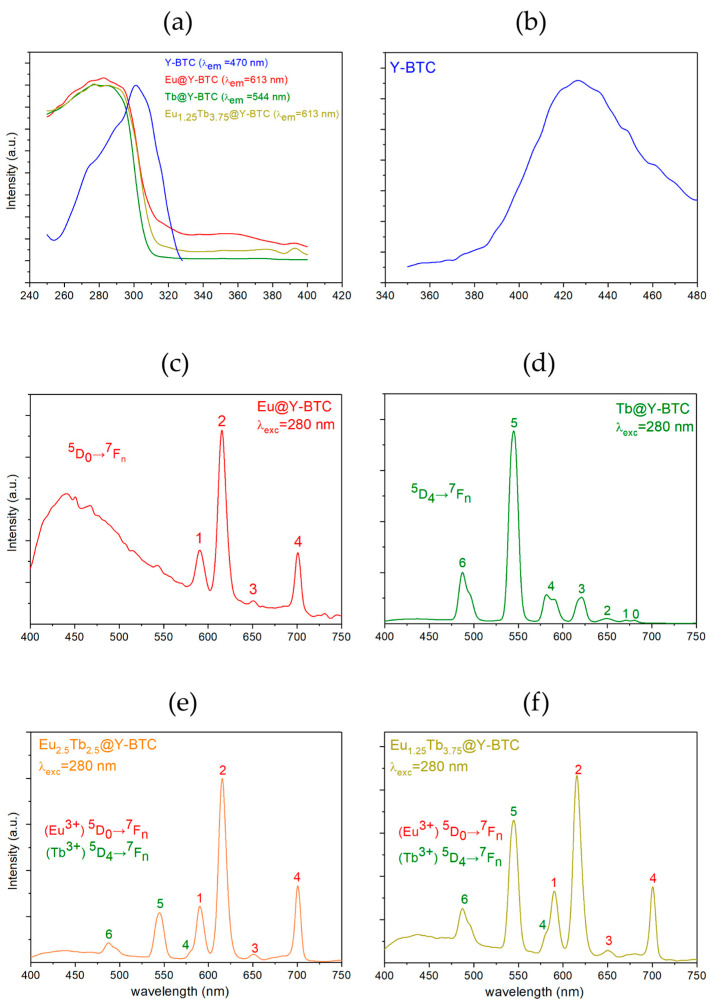
(**a**) Excitation spectra of **Y-BTC** and **Ln@Y-BTC** samples; emission profiles from (**b**) **Y-BTC**, (**c**) **Eu@Y-BTC**, (**d**) **Tb@Y-BTC**, (**e**) **Eu_2.5_Tb_2.5_@Y-BTC,** and (**f**) **Eu_1.25_Tb_3.75_@Y-BTC** upon excitation at 280 nm.

**Figure 4:**
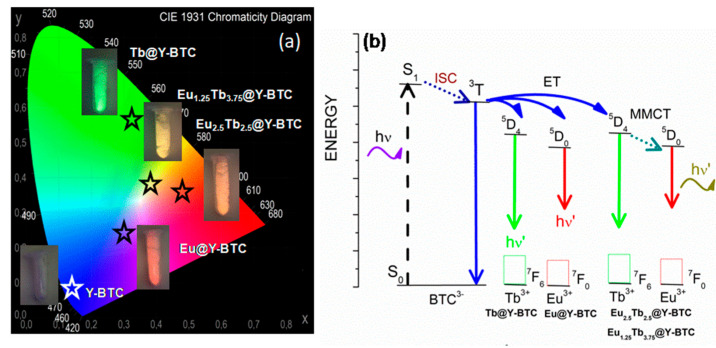
(**a**) CIE diagram showing the color coordinates; (**b**) the proposed Jablonski diagram for the compounds reported herein.

Photoluminescence (PL) characterization includes key parameters [42] such as (a) PL spectra; (b) quantum yields; and/or (c) observed luminescence lifetimes (τ_obs_). Moreover, the precise quantification of the emitting light is essential for the development of optical devices used in electronics, and chemical and physical sensors [43]. In this context, the room-temperature solid-state photoluminescence properties of **Y-BTC** and **Ln@Y-BTC** compounds were explored. When the **Y-BTC** is excited at 280 nm, an emission is achieved that corresponds to a broad band located at 430 nm (Figure 3b). This transition is dominant and responsible for the purplish-blue emission, as confirmed by the calculated CIE(x,y) chromaticities coordinates (see Figure 4a). Additionally, the τ_obs_ of emission is 0.0216 ms, the smallest value from the sets, which is consistent with typical values for organic molecules.

The photoluminescence behavior of the doped samples, as expected from the lanthanide 4f-transitions, was characterized by recording excitation profiles by monitoring the 4f emitting lines: 613 nm for Eu^3^⁺ and 544 nm for Tb^3^⁺. These profiles revealed an intense absorption transition centered at 280 nm (Figure 3a). This transition is seen as a typical broadband, is related to π*← π or π*← n transitions from the organic BTC^3−^ moieties and was selected for setting the excitation wavelength for the sample sets. Upon ligand sensitization (τ_exc_ = 280 nm) **Eu@Y-BTC** exhibits a dual emission from the BTC^3−^ ligand at 439 nm due to π* → n/π* → π transitions, accompanied by fine 4f-transitions centered at ^5^D_0_ → ^7^F_n_ (n = 1–4) transitions observed at 589, 616, 652, and 700 nm, respectively (Figure 3c). The combination of the mentioned transitions led to a purplish-pink emission (Figure 4a). The ^5^D_0_ → ^7^F_2_ transition was the most intense with a τ_obs_ of 0.28 ms. In most cases, the hypersensitive ^5^D_0_ → ^7^F_2_ transition is the most intense transition responsible for the typical red emission in Eu-containing compounds [33].

Similarly, **Tb@Y-BTC**, upon excitation at 280 nm, displayed the characteristic, ^5^D_4_ → ^7^F_n_ (n = 6–0) transitions centered at 487, 544, 580, 622, 649, 673 and 682, which are attributed to Tb^3+^ 4f*-4f transitions, respectively. In this case, the strongest emission is associated with the ^5^D_4_ → ^7^F_5_ transition (544 nm), which is responsible for the bright green emission (see Figure 3d) [44]. Also, the lifetime value was calculated as 1.36 ms, being the longest among the studied samples in this work. For the **Eu_2.5_Tb_2.5_@Y-BTC** sample, upon 280 nm excitation, two sets of lanthanide transitions belonging to Eu^3+^ and Tb^3+^ were observed, yielding an orange-pink emission (Figure 3e). The dominant transition is the ^5^D_0_ → ^7^F_2_ from Eu^3+^ ions, principally due to the efficient energy migration from the emitting level of Tb^3+^ to Eu^3+^. In this sense, the close proximity of the excited levels for both lanthanides makes a metal-to-metal charge transfer feasible [45]. The *τ*_obs_ values for the **Eu_2.5_Tb_2.5_@Y-BTC** sample were calculated from decay data, being 0.24 ms for Eu^3+^ and 0.28 ms for Tb^3+^. These lifetime values reinforce the mechanism of energy transfer among the lanthanide ions.

In the **Eu_1.25_Tb_3.75_@Y-BTC** sample, a higher Tb^3^⁺ content resulted in an increase in the intensity of the ^5^D_4_ → ^7^F_5_ (Tb^3^⁺) transition (Figure 3f). However, the emission from europium ions remained more prominent, leading to a yellow color. The corresponding τ_obs_ values were 0.39 for Eu^3+^ ions and 0.64 ms for Tb^3+^, also reinforcing the mechanism of metal-to-metal charge transfer among both metal ions. Additionally, according to the proposed Jabloski diagram (Figure 4b), the emissive level of terbium is higher than that of europium, therefore, it is reasonable to assume a depopulation from the ^5^D_4_ (Tb^3+^) levels to partially feed the ^5^D_0_ (Eu^3+^) levels. Previously, we explored the optical response of RGB-SURMOFs (Red-Green-Blue) devices based on the **Gd-BTC** structure combined with **Eu-** and **Tb-BTC** nanolayers with a heteroepitaxial geometry [35] In that study, an efficient energy transfer was confirmed involving BTC^3−^ → Tb^3+^ → Eu^3+^ energy migration, resulting in white light emission. To gain deeper insights into the energy transfer processes in the mixed samples, the energy transfer efficiencies (η_T_) from Tb^3+^ to Eu^3+^ were calculated using the following formula (Equation (1)) [46]:

(1) $$\eta_{Tb\to Eu}=1-\left(\frac{\tau }{\tau_{0}}\right)$$

where $\eta_{Tb\to Eu}$ is the energy transfer efficiency and τ_0_ and τ are the observed lifetimes of Tb^3+^ ions in the absence and presence of Eu^3+^ ions, respectively. Thus, the relationship between the energy-transfer efficiency and activator concentration of Eu^3+^ ions can be analyzed. The value of $\eta_{Tb\to Eu}$ reaches a maximum of 82% in the **Eu_2.5_Tb_2.5_@Y-BTC** sample and a value of 53% in **Eu_1.25_Tb_3.75_@Y-BTC**.

High-quality light performance requires the calculation of the Commission International de l’Eclairage (CIE) x,y chromaticity coordinates and the correlated color temperature (CCT), which are critical parameters for solid-state lighting applications. Quantifying the color emission of different luminescent sources allows their comparison by studying the corresponding light-emitting performance. In this context, the color coordinates are usually calculated using the CIE x,y chromaticity system and plotted in a two-dimensional diagram, providing a visual representation of emission features. The color emission of **Y-BTC** and **Ln@Y-BTC** was quantified as shown in Figure 4a and detailed in Table 1. Moreover, the lanthanide-doped samples showed CCT values of 13,546.5, 5770.3, 2028.6, and 3884.2 K, respectively, matching the human eye-friendly application range and photometry implementations [37]. To further assess the quality of the emitted color, the color purity of the emitted color in all the samples was determined through the following equation (Equation (2)) [47]:

(2) $$color\;purity=\sqrt{\frac{{\left(x-x_{s}\right)}^{2}+{\left(y-y_{s}\right)}^{2}}{{\left(x_{d}-x_{s}\right)}^{2}+{\left(y_{d}-y_{s}\right)}^{2}}}\;\times \;100$$

where x and y represent the CIE coordinates of the entire spectrum. x_s_ and y_s_ denote the CIE coordinates of the standard illuminants of white light; and x_d_ and y_d_ stand for the CIE coordinates of the dominant wavelength. Notably, **Y-BTC** and **Tb@Y-BTC** exhibited the highest color purity values (see Table 1). However, in some doped samples, the primary transitions were influenced by additional spectral features, affecting color purity. For instance, **Eu@Y-BTC** presented emission contributions not only from Eu^3+^ ions but also from the BTC^3−^ ligand, reducing the purity of the red emission. Similarly, in co-doped samples, the red emission from Eu^3+^ was affected by the intense green emission from Tb^3+^ ions, which impacted the overall emission profile and the perceived color.

For an in-depth evaluation of the luminescence efficiency of the Eu-containing compounds, the intrinsic quantum yields (Q_Eu_) were calculated (see Table 2). This parameter, along with the efficiency of sensitization, determines the overall luminescence quantum yield (Q_Y_). Assuming that non-radiative and radiative processes are essentially involved in the depopulation of the ^5^D_0_ state, the Q_Eu_ can be expressed as follows:

(3) $$Q_{Eu}=\frac{k_{rad}}{\left(k_{rad}+k_{nrad}\right)}$$

Thus, Q_Eu_ of the luminescence expresses how well the radiative processes (k_rad_) compete with the non-radiative processes (k_nrad_) described by Equation (3). In general, non-radiative contributions include back-energy transfer to the sensitizer, electron transfer quenching, and quenching by matrix vibrations. Additionally, vibrations commonly found in organic molecules (C–H, O–H, N–H) can contribute to k_nrad_ [48]. The radiative contribution k_rad_ can be estimated from the equation:

(4) $$k_{rad}=\frac{1}{\tau_{rad}}$$

The radiative lifetime τ_rad_ can be approximated for Eu(III) by the Equation (5) [49]:

(5) $$k_{rad}=A_{MD,0}.n^{3}.\left(\frac{I_{tot}}{I_{MD}}\right)$$

Here, A_MD,0_ is the spontaneous emission probability of the magnetic dipole ^5^D_0_ → ^7^F_1_ transition (14.65 s^−1^), *n* is the refractive index (being 1.5 for solids), I_tot_ is the total integrated emission of the ^5^D_0_ → ^7^F_J_ (J = 0–6) transitions and I_MD_ is the integrated emission of the ^5^D_0_ → ^7^F_1_ transition. If the τ_rad_ is known, Q_Eu_ can be calculated using the τ_obs_. Based on Equations (3) and (5), Q_Eu_ can be calculated as:

(6) $$Q_{Eu}=\frac{\tau_{obs}}{\tau_{rad}}$$

Additionally, by knowing the τ_obs_ and τ_rad_ it is possible to determine the overall rate of non-radiative deactivation (k_nrad_). Hence, the radiative lifetime is an important parameter for the photophysical description of lanthanide luminescence. The photophysical characterization for the Eu-doped MOFs reported here is summarized in Table 2. According to the PL parameters exhibited in Table 2, it is possible to highlight the promising emission properties of high europium quantum yield and lifetime values in **Eu_1.25_Tb_3.75_@Y-BTC**.

## 4. Sensing Assays

MOFs have been widely employed in various implementations, including powders, thin films, and composites, for sensing applications. They have demonstrated effectiveness in detecting organic solvents [50], VOCs [51], and metal ions [52]. Additionally, the detection of hazardous analytes, such as explosive molecules [53], is a growing area of interest for MOF-based sensors.

Ln-MOFs, particularly those containing Eu^3+^ and Tb^3+^, have gained attention due to their hypersensitive transitions, which provide PL properties that are highly responsive to changes in the chemical and physical environment [54,55]. This feature is relevant for sensor design, either in powder form or anchored into solid substrates. The dual-emission capability of Eu^3+^ and Tb^3+^ ions presents a unique opportunity for chemosensing performance.

**Figure 5:**
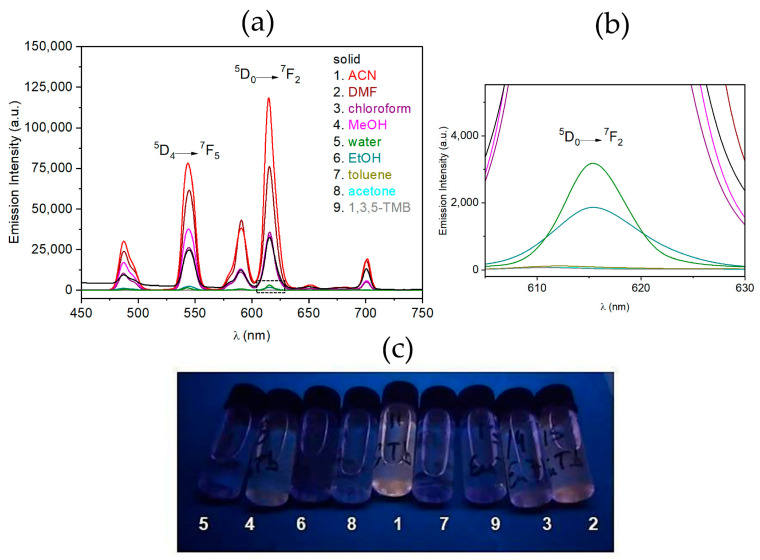
(**a**) PL spectra of **VOC@Eu_1.25_Tb_3.75_@Y-BTC** suspensions recorded at room temperature (λ_exc_ = 280 nm); (**b**) inset into the ^5^D_0_ → ^7^F_2_ signal and (**c**) their color effects under UV light exposure.

The chemosensing performance of **Eu_1.25_Tb_3.75_@Y-BTC** was investigated by measuring the PL in the presence of three protic solvents (water, methanol, and ethanol) and six aprotic solvents (DMF, chloroform, toluene, 1,3,5-TMB, and acetone). Upon excitation at 280 nm, the typical Eu^3+^ and Tb^3+^transitions are observed in the emission spectra (Figure 5), the ^5^D_0_ → ^7^F_2_ (Eu^3+^) and ^5^D_4_ → ^7^F_5_ (Tb^3^⁺) transitions being the most intense.

Comparing the emission profiles of the solid-state samples (black trace) with those of the VOC-exposed (**VOC@Eu_1.25_Tb_3.75_@Y-BTC**), significant variations were observed. Notably, there are certain VOCs, such as ACN, DMF, chloroform, and MeOH, that enhance the ^5^D_0_ → ^7^F_2_ transition intensity by 274, 143, 15, and 6.5%, respectively. In contrast, EtOH, toluene, 1,3,5-TMB, and acetone produce a significant quenching effect, which is detectable to the “naked eye”.

The quenching efficiency (QE) of VOCs was calculated from Equation (7) [56]:

(7) $$QE\%=\left(I_{0}-I\right)/I\;\cdot 100$$

where I_0_ and I represent the emission intensity values in the absence and presence of the VOC, respectively. The calculated QE values for water, EtOH, toluene, acetone, and 1,3,5-TMB were 89, 94, 99.6, 99.7, and 99.8%.

These findings demonstrate the potential of **Eu_1.25_Tb_3.75_@Y-BTC** as a highly sensitive and selective luminescent sensor for VOC detection, offering promising applications in environmental monitoring and industrial settings.

**Figure 6:**
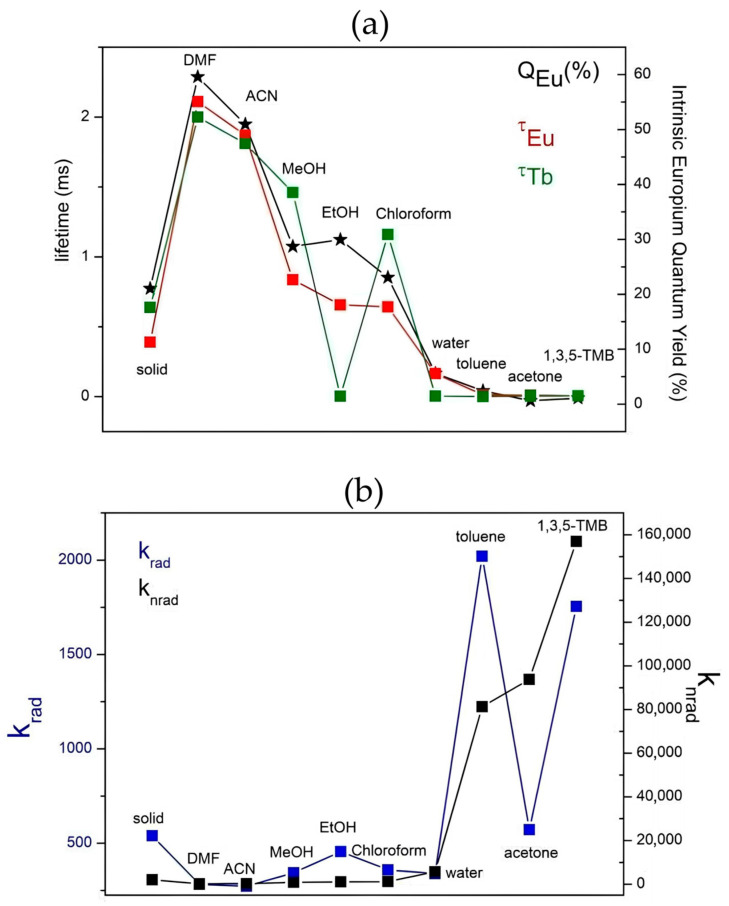
(**a**) Q_Eu_ (%) and lifetime values; (**b**) k_rad_ and k_nrad_ constants of **VOC@Eu_1.25_Tb_3.75_@Y-BTC** systems.

Understanding the intricate interplay between the host framework and guest VOC molecules is essential for optimizing the sensing performance and expanding the potential applications of luminescent MOF-based sensors. To achieve a comprehensive understanding of the PL behavior, it is essential to analyze additional PL parameters such as k_rad_, k_nrad_, and k_exp_ (k_exp_ = k_rad_ + k_nrad_) constants, Q_Eu_ and τ_obs_ (Figures S4 and S5 and Table S1). Figure 6 presents the PL parameters of **VOC@Eu_1.25_Tb_3.75_@Y-BTC** systems, offering insights into the interaction dynamics between the analytes and lanthanide ions.

Analyte–lanthanide ion interactions may be inferred from the determination of energy transfer efficiency within the frame of Förster’s dipole–dipole mechanism that supports the quenching effects [57]. In this context, Equation (1) is also useful to estimate the efficiency of transfer between the donor (Eu or Tb) and the acceptor (VOCs) as follows:

(8) $$\eta_{Eu\to VOC}=1-\left(\frac{\tau_{VOC}}{\tau_{s}}\right)$$

(9) $$\eta_{Tb\to VOC}=1-\left(\frac{\tau_{VOC}}{\tau_{s}}\right)$$

The calculated η_Eu→VOC_ were 57.7, 96.9, 97.28 and 98.4% in water, toluene, acetone and 1,3,5-TMB. The corresponding η_Tb→VOC_ values were 99.45, 99,4, 99.8, 99.4, and 99.1% for EtOH, water, toluene, acetone, and 1,3,5-TMB. These values indicate a stronger sensitization of Tb^3+^ with respect to Eu^3+^ ions in the presence of the mentioned VOCs.

The quenching mechanism mediated by coupling vibrations is based on the capability of certain atomic groups to dissipate part of the lanthanide energy. This effect can be quantitatively assessed by the so-called “quantum numbers” [49], which represent the number of times such vibrational stretching matches up with the 4f electronic transition. In the present study, the energy of the Eu^3+^ hypersensitive ^5^D_0_ → ^7^F_2_ transition (16,313 cm^−1^) corresponds to approximately 4.4 times the energy of -OH (3650 cm^−1^), 6 times that of -CH (2960 cm^−1^), and 9.5 times that of -C=O (1680 cm^−1^) [58].

Similarly, the energy of the ^5^D_4_ → ^7^F_5_ transition of Tb^3+^ (18,382 cm^−1^) corresponds to approximately 5, 6.2, and 11 times the vibrational energies of -OH, -CH, and -C=O groups, respectively. Since the lower the quantum number is, the higher the quenching efficiency, hydroxyl (-OH) groups are more efficient in luminescence attenuation compared to other organic groups. Interestingly, this fact aligns more closely with the calculated energy transfer values for η_Tb→VOC_ than for η_Eu→VOC,_ highlighting a differential response of the lanthanide ions to various molecular environments.

Comparing the k_nrad_ observed in the **Eu_1.25_Tb_3.75_@Y-BTC** solid, the increased values in the presence of toluene, 1,3,5-TMB, and acetone suggest that the quenching of Eu^3+^ emission predominantly occurs due to -C=O and -CH groups rather than -OH groups, via non-radiative pathways. Due to the porous nature of the MOF-76 structure [59], these results imply a size–analyte-dependence quenching mechanism facilitated by interactions with lanthanide centers through 6.6 × 6.6 Å^2^ 1D-channels. A similar phenomenon has been previously reported in **Eu-BTC** frameworks for agrochemical detection [60].

Also, DMF, water, and MeOH, which are commonly coordinated to lanthanide ions in MOF-76, further reinforce the hypothesis that analyte–MOF interactions enhance the sensitization process [52].

Additionally, the solvent-dependent luminescence of Eu^3^⁺ and Tb^3^⁺ was analyzed by evaluating competitive pathways between energy transfer and photoinduced electron transfer (PET). Frontier molecular orbital energies (HOMO–LUMO) of the ligand triplet excited state (T1) were calculated in nine analyte-solvents (Table 3).

The values in Table 3 were compared to the excited-state energies of Eu^3^⁺ (^5^D_0_: ~2.0 eV) and Tb^3^⁺ (^5^D_4_: ~2.5–2.7 eV), as well as their redox potentials. The experimental trends— enhanced Eu^3^⁺ emission in ACN, DMF, methanol, and chloroform versus quenching in acetone, ethanol, water, toluene, and 1,3,5-TMB—are explained below and extended to Tb^3^⁺ ions.

In ACN, DMF, and methanol, the ligand’s HOMOα (−4.85 to −4.78 eV) lies below the Eu^3^⁺ excited-state reduction potential (−4.7 eV), suppressing PET (Table 4). The large T_1_ energy gap (6.2–6.3 eV) exceeds both Eu^3^⁺ ^5^D_0_ and Tb^3^⁺ ^5^D_4_ energies, enabling efficient Dexter-type energy transfer. For Tb^3^⁺, this alignment suggests strong sensitized emission, assuming minimal non-radiative decay. Chloroform shows distinct behavior; its HOMOα (−3.05 eV) aligns with Eu^3^⁺ redox potential, but the high LUMOα (2.99 eV) elevates T_1_ energy (6.04 eV), favoring PET for both Eu^3^⁺ and Tb^3^⁺.

In acetone and ethanol, HOMOα (−4.61 to −4.69 eV) approaches the Eu^3^⁺ ^5^D_0_ reduction potential (−4.7 eV), enabling PET (Table 5). For Tb^3^⁺, T_1_ energy (6.2 eV) exceeds ^5^D_4_ (~2.5–2.7 eV), but PET may compete if the Tb^3^⁺ excited-state reduction potential is less negative than that of Eu^3^⁺. Toluene and 1,3,5-TMB exhibit extreme PET due to high HOMOα (−1.03 to −0.64 eV). For Tb^3^⁺, PET likely dominates despite sufficient T_1_ energy (5.2–5.7 eV), as the HOMOα alignment overrides energy transfer. Finally, water suppresses emission via solvent polarity effects (e.g., O-H vibrational quenching), destabilizing the T_1_ state for both Eu^3^⁺ and Tb^3^⁺, despite unfavorable PET thermodynamics.

A comparative analysis between Eu^3^⁺ and Tb^3^⁺ reveals the following key findings. First, regarding energy transfer efficiency, the T_1_ energy level (~6 eV) is sufficient to sensitize both ions; however, it is less optimal for Tb^3^⁺ due to the smaller energy gap between the T_1_ and ^5^D_4_ states compared to the T_1_ and ^5^D_0_ states in Eu^3^⁺, thereby reducing the efficiency of Förster resonance energy transfer (FRET). Second, in terms of PET competition, the higher ^5^D_4_ energy of Tb^3^⁺ results in a less negative reduction potential. Should the excited-state reduction potential of Tb^3^⁺ exceed −4.7 eV, PET could compete with other processes in solvent–analytes such as acetone. Third, analyte effects play a significant role: non-polar solvents (e.g., toluene and 1,3,5-TMB) favor PET for both ions, whereas polar solvents like water predominantly induce non-radiative decay pathways, irrespective of the ion species.

These results provide a solid foundation for the development of VOC sensors based on lanthanide coordination polymers, especially for vapor detection devices for air quality monitoring and use in industrial settings.

## 5. Conclusions

In summary, the photoluminescence analysis of **Eu_1.25_Tb_3.75_@Y-BTC** demonstrates its potential as a highly sensitive and selective sensor for VOC detection. The observed quenching and enhancement effects in the presence of different analytes highlight the significant role of analyte size, functional group interactions, and the porous nature of the MOF-76 framework in modulating the photoluminescence response. The study reveals a size-dependent quenching mechanism, where the analyte diffusion through the 1D channels and the coordination of protic solvents to lanthanide centers significantly influence the sensing performance.

Despite the advances in VOC detection, traditional methods remain limited by high costs, complex instrumentation, and slow response times, emphasizing the need for efficient, recyclable, and easily deployable sensing platforms. The enhanced sensitivity of Tb^3^⁺ over Eu^3^⁺ ions towards specific VOCs underscores the potential of dual-emission sensing, offering improved selectivity and real-time monitoring capabilities. Furthermore, the structural stability and recyclability of the Ln@Y-BTC material make it a strong candidate for real-world applications, particularly in air quality monitoring and industrial safety. Understanding the interplay between the host framework and guest VOC molecules is crucial for optimizing sensing performance and broadening the applicability of luminescent MOF-based sensors.

This work not only expands the understanding of lanthanide-based luminescent MOFs for VOC detection but also establishes a foundation for further research aimed at fine-tuning MOF structures for enhanced sensitivity, selectivity, and practical implementation in portable sensing technologies.

## Figures and Tables

**Table 1:** CIE chromaticity, CCT values, and calculated color purity from all the compounds.

Sample	CIE Chromaticity from the Entire Spectrum		CCT (K)	CIE Chromaticity from the Dominant Wavelength		Color Purity (%)
	x	y		x_d_	y_d_	
**Y-BTC**	0.144	0.07	1937.4	0.17	0.006	89.97
**Eu@Y-BTC**	0.281	0.25	13,546.5	0.68	0.31	21.28
**Tb@Y-BTC**	0.319	0.545	5770.3	0.26	0.73	55.75
**Eu_2.5_Tb_2.5_@Y-BTC**	0.474	0.354	2028.6	0.68	0.31	45.11
**Eu_1.25_Tb_3.75_@Y-BTC**	0.384	0.373	3884.2	0.68	0.31	25.76

**Table 2:** Photophysical parameters of the europium-based MOFs.

Compound	I_tot_/I_MD_	*τ*_rad_/ms	k_rad_/s^−1^	k_exp_/s^−1^	k_nrad_/s^−1^	*τ*_obs_/ms	Q_Eu_ (%)
**Eu@Y-BTC**	14.52	1.39	718.1	3571.42	2853.32	0.28	20.1
**Eu_2.5_Tb_2.5_@Y-BTC**	9.58	2.11	474.05	3508.77	3034.71	0.285	13.51
**Eu_1.25_Tb_3.75_@Y-BTC**	10.9	1.85	539.33	2564.1	2024.76	0.39	21.03

**Table 3:** Energy levels (eV) of the ligand in various analytes. This table presents the HOMO and LUMO energies for both α and β electrons, along with their respective energy gaps (ΔHLα/β).

Analyte	HOMO_a_	LUMO_a_	HOMO_b_	LUMO_b_	ΔHL_a_	ΔHL_b_	ΔHL_tot._
ACN	−4.8533	1.4235	−8.0813	−2.0437	6.2771	6.0392	2.8091
DMF	−4.8173	1.3894	−8.1367	−2.1077	6.2064	6.0303	2.7096
Chloroform	−3.0479	2.988	−6.3256	−0.338	6.0375	5.988	2.7096
Methanol	−4.7804	1.425	−8.0983	−2.0716	6.206	6.0294	2.7093
Water	−5.0072	1.2801	−8.2273	−2.1854	6.2876	6.0433	2.8214
Ethanol	−4.6893	1.515	−8.0041	−1.9833	6.2046	6.0227	2.7063
Toluene	−1.0325	4.6158	−4.2754	1.6907	5.648	5.9665	2.7232
Acetone	−4.6073	1.5966	−7.9209	−1.9015	6.204	5.9204	2.706
1,3,5-TMB	−0.6353	4.5709	−4.0302	2.2521	5.2067	6.2826	2.8865

**Table 4:** Group A solvents: enhanced emission (energy transfer dominates).

Analyte	HOMOα (eV)	LUMOα (eV)	T_1_ Energy (eV)	Relevance to Tb^3+^
ACN	−4.85	1.42	6.27	“T1 >> Tb^3^⁺ ^5^D_4_ (~2.5–2.7 eV); efficient energy transfer”
DMF	−4.82	1.39	6.21	“Similar to ACN”
Methanol	−4.78	1.43	6.21	“enough T_1_ energy for Tb^3^⁺ sensitization”
Chloroform	−3.05	2.99	6.04	“High T_1_ ensures PET despite moderate HOMOα”

**Table 5:** Group B solvents: quenched emission (PET or analyte effects).

Solvent	HOMOα (eV)	LUMOα (eV)	T_1_ Energy (eV)	Primary Quenching Mechanism	Relevance to Tb^3+^
Acetone	−4.61	1.6	6.21	“PET (HOMOα ≈ −4.7 eV)”	“PET competes with energy transfer (T_1_ > ^5^D_4_)”
Ethanol	−4.69	1.52	6.21	“Marginal PET competition”	“Similar to acetone”
Water	−5.01	1.28	6.29	“Solvent-induced non-radiative decay”	“Polarity disrupts T_1_ state for both ions”
Toluene	−1.03	4.62	5.65	“Strong PET (high HOMOα)”	“PET dominates despite T_1_ > ^5^D_4_”
1,3,5-TMB	−0.64	4.57	5.21	“Extreme PET (very high HOMOα)”	“PET overrides energy transfer”

## Data Availability

The original contributions presented in this study are included in the article/supplementary material. Further inquiries can be directed to the corresponding author.

## Supplementary Materials

The following supporting information can be downloaded at: https://www.mdpi.com/article/10.3390/polym17091135/s1. Detailed theoretical calculations such as Optimized Cartesian coordinates of the BTC ligand in the different analytes; Table S1. Quasiharmonic DFT thermodynamic properties; Figures S1–S9 Energy diagram and graphical representation of the ligand frontier orbitals in the analytes; Table S2. Computed absorption energies, excitation state character and transition weight; Figure S10: PXRD patterns of thermal analysis residue compared with the simulated Y₂O₃ pattern; Figure S11. FTIR spectra of **Y-BTC** and **Ln@Y-BTC**; Figure S12. Decay profiles of the **Ln@Y-BTC** solid compounds; Figure S13. Terbium ^5^D_4_ decay profiles of **VOC@Eu_1.25_Tb_3_**_.75_ suspensions; Figure S14. Europium ^5^D_0_ decay profiles of **VOC@Eu_1.25_Tb_3.75_** suspensions; Table S3. Photophysical parameters of **VOC@Eu_1.25_Tb_3.75_** suspensions; Table S4. Eu and Tb content in co-doped samples determined by ICP-AESP.
