# Supplementary material for: Engineering Photoluminescence of Lanthanide Doped Yttrium-MOF-76 for Volatile Organic Compound Sensing
Source: Polymers (Basel). 2025 Apr 22;17(9):1135. doi: 10.3390/polym17091135 (PMC12073188; doi:10.3390/polym17091135)
Supplement: Supplementary file 1 [file polymers-17-01135-s001.zip › polymers-3477499-supplementary.pdf]

Electronic Supporting Information

Engineering photoluminescence of lanthanide doped yttrium-MOF-76 for volatile organic compound sensing

Oswaldo Rosas R.[1,2], Mariana Hamer[3], Héctor A. Baldoni[1,4], Maya Boone[5], Rik Van Deun[5], Germán E. Gomez*[1,2]

1 Universidad Nacional de San Luis, Facultad de Química, Bioquímica y Farmacia, Área de Química General e Inorgánica "Dr. G. F. Puelles", Ejército de los Andes 950 (5700), San Luis, Argentina.

2 Instituto de Investigaciones en Tecnología Química (INTEQUI), Almirante Brown 1455 (5700), San Luis, Argentina. gegomez@unsl.edu.ar

3 Universidad Nacional de General Sarmiento, Área Química, Instituto de Ciencias, CONICET. J. M. Gutiérrez 1150, Los Polvorines, Buenos Aires, Argentina.

4 Instituto Multidisciplinario de Investigaciones Biológicas de San Luis, IMIBIO-SL, CONICET-UNSL, Av. Ejército de los Andes 950 (5700), San Luis, Argentina

5 L3 – Luminescent Lanthanide Lab, Ghent University, Department of Chemistry, Krijgslaan 281, Building S3, 9000 Gent, Belgium.

* Correspondence: gegomez@unsl.edu.ar

Theoretical calculations section

Listing S1. Optimized Cartesian coordinates of the ligand in the analyte acetonitrile.

```
1\1\ginc-bdw03\fopt\uwb97xd\6-311++g(d,p)\c9h3o6(3-,3)\++++++++\08-mar
-2025\0\\#p wb97xd/6-311++g(d,p) opt=tight guess=read geom=check scrf=
(solvent=acetonitrile) int=ultrafine nosymm freq=noraman pop=full\\1,3
,5-btc\\-3,3\c,-0.0076347431,-1.4334806333,-0.0278903172\c,1.256935469
7,-0.6726295238,-0.1046428259\c,-1.2597911452,-0.6792158626,-0.0911215
554\c,1.2704344177,0.6683084265,0.0203575684\h,2.1788475263,-1.2208531
081,-0.2451651716\c,-0.0029151691,-2.9145027314,0.1090148061\c,-1.2730
601351,0.6620880008,0.0266034927\h,-2.1848880256,-1.2272808091,-0.2132
643855\c,0.0060553039,1.3769361479,0.2042141987\c,2.5732518417,1.46300
55065,-0.0509676088\o,-1.1209600364,-3.5031280247,0.0785219734\o,1.121
2606238,-3.4780768288,0.2389988939\c,-2.570055348,1.4587555553,-0.0443
793213\h,0.0042512124,2.4372251654,0.4030808312\o,2.4586624423,2.71428
95981,-0.0537255936\o,3.6487208456,0.818721784,-0.1040979752\o,-2.4489
644808,2.7099498009,-0.0690003329\o,-3.6501506001,0.8198875362,-0.0765
36677\\version=es64l-g16revc.01\hf=-796.4327181\s2=2.009983\s2-1=0.\s2
a=2.000062\rmsd=7.041e-09\rmsf=6.393e-06\dipole=-0.013706,0.0716286,-0
.0349659\quadrupole=-29.2246122,-28.1661748,57.390787,-0.1209343,-0.44
85917,4.1613089\pg=c01 [x(c9h3o6)]\\@
```

Listing S2. Optimized Cartesian coordinates of the ligand in the analyte n,n-dimethylformamide.

```
1\1\ginc-bdw03\fopt\uwb97xd\6-311++g(d,p)\c9h3o6(3-,3)\++++++++\08-mar
-2025\0\\#p wb97xd/6-311++g(d,p) opt=tight guess=read geom=check scrf=
(solvent=n,n-dimethylformamide) int=ultrafine nosymm freq=noraman pop=
full\\1,3,5-btc\\-3,3\c,0.0542562053,-1.4340648141,0.0149676078\c,1.24
40924467,-0.7170925067,0.0095266153\c,-1.2484218329,-0.6660077878,0.04
01396669\c,1.2698568466,0.6675725675,-0.0012968843\h,2.1814241436,-1.2
597434538,0.0093823678\c,0.0344204569,-2.9337397346,-0.0283352879\c,-1
.2517236683,0.7191644634,0.0042161604\h,-2.1722307634,-1.2229769857,0.
0824418849\c,-0.0471955129,1.410606615,-0.0280004425\c,2.5588844361,1.
4385350993,0.0103536297\o,-1.0977030296,-3.4751960912,-0.1606777752\o,
1.1343881445,-3.5413347137,0.0648604509\c,-2.5813639812,1.4900242971,0
.0056862035\h,-0.0283424249,2.4890094869,-0.0666014918\o,2.4568160107,
2.6960692716,0.0277836699\o,3.6380635676,0.7879810298,0.0033078045\o,-
2.5126226515,2.7391321984,-0.0862527736\o,-3.6325983934,0.8120610585,0
.0984985937\\version=es64l-g16revc.01\hf=-796.4335728\s2=2.067798\s2-1
=0.\s2a=2.002063\rmsd=4.485e-09\rmsf=6.088e-06\dipole=-0.0347325,0.002
639,0.0475828\quadrupole=-29.4637882,-28.2531058,57.716894,1.0110062,-
0.6246183,-0.559756\pg=c01 [x(c9h3o6)]\\@
```

Listing S3. Optimized Cartesian coordinates of the ligand in the analyte chloroform.

```
1\1\ginc-bdw06\fopt\uwb97xd\6-311++g(d,p)\c9h3o6(3-,3)\++++++++\08-mar
-2025\0\\#p wb97xd/6-311++g(d,p) opt=tight guess=read geom=check scrf=
(solvent=chloroform) int=ultrafine nosymm freq=noraman pop=full\\1,3,5
-btc\\-3,3\c,0.0490537474,-1.4357462126,0.017751537\c,1.2454468828,-0.
7139899285,0.0131150861\c,-1.2547890588,-0.6660385044,0.0155431843\c,1
.2730684804,0.6654710975,0.0005139891\h,2.1808607805,-1.2615351009,0.0
134195623\c,0.0361807415,-2.9378285225,-0.0097953456\c,-1.2587824669,0
.7142076911,0.0059696246\h,-2.1792766059,-1.2249949792,0.0141212595\c,
-0.0458804106,1.4083035122,0.0002606316\c,2.5672206106,1.4400323883,-0
.0137926579\o,-1.0932643124,-3.4902619498,-0.1213463708\o,1.1422783864
,-3.5359490103,0.0789521703\c,-2.5882460646,1.4937518467,0.0084261629\
h,-0.030498269,2.4880397913,-0.0042486588\o,2.4622226419,2.6973181452,
-0.0304075871\o,3.6465770566,0.7905280784,-0.0085486946\o,-2.503747602
6,2.7455128353,-0.0012259534\o,-3.6484245373,0.8231788222,0.0212920603
\\version=es64l-g16revc.01\hf=-796.332613\s2=2.066149\s2-1=0.\s2a=2.00
197\rmsd=8.090e-09\rmsf=2.228e-05\dipole=-0.0377481,0.0163933,0.049864
```

```
6\quadrupole=-29.4849723,-28.2601634,57.7451357,1.0553538,-0.2940343,-
0.147312\pg=c01 [x(c9h3o6)]\\@
```

Listing S4. Optimized Cartesian coordinates of the ligand in the analyte methanol.

```
1\1\ginc-bdw03\fopt\uwb97xd\6-311++g(d,p)\c9h3o6(3-,3)\++++++++\08-mar
-2025\0\\#p wb97xd/6-311++g(d,p) opt=tight guess=read geom=check scrf=
(solvent=methanol) int=ultrafine nosymm freq=noraman pop=full\\1,3,5-b
tc\\-3,3\c,0.0541439492,-1.434121852,0.0149148328\c,1.2440972956,-0.71
7008668,0.0095886466\c,-1.2486129897,-0.6660426691,0.0386712075\c,1.26
991121088,0.6675417788,-0.0011331428\h,2.181393994,-1.2597530813,0.0094
566168\c,0.0345259365,-2.9338793108,-0.0272274584\c,-1.2519015546,0.71
90420748,0.0042794765\h,-2.1724876958,-1.2230898674,0.0786722733\c,-0.
0472021349,1.4105824422,-0.0264272388\c,2.5590723456,1.4385813705,0.00
91398373\o,-1.0975377863,-3.4757239398,-0.1585582562\o,1.1346845272,-3
.5411564766,0.0659621142\c,-2.5815166405,1.4901458647,0.0058024454\h,-
0.0284647749,2.4890442329,-0.0632242381\o,2.4570096933,2.6961420468,0.
0246690732\o,3.6382251052,0.7879919341,0.0028251045\o,-2.5122270228,2.
7395555968,-0.0815761919\o,-3.6331143559,0.8121485237,0.0941648981\\ve
rsion=es64l-g16revc.01\hf=-796.4314893\s2=2.067783\s2-1=0.\s2a=2.00206
2\rmsd=5.572e-09\rmsf=6.014e-06\dipole=-0.0351328,0.0031206,0.0474755\
quadrupole=-29.4646122,-28.2590093,57.7236215,1.0116226,-0.6102032,-0.
5349917\pg=c01 [x(c9h3o6)]\\@
```

Listing S5. Optimized Cartesian coordinates of the ligand in the analyte water.

```
1\1\ginc-bdw05\fopt\uwb97xd\6-311++g(d,p)\c9h3o6(3-,3)\++++++++\08-mar
-2025\0\\#p wb97xd/6-311++g(d,p) opt=tight guess=read geom=check scrf=
(solvent=water) int=ultrafine nosymm freq=noraman pop=full\\1,3,5-btc\
\-3,3\c,-0.006292171,-1.4335205115,-0.0331863296\c,1.2571739356,-0.673
129405,-0.1115396718\c,-1.2594253298,-0.6787346359,-0.0998081078\c,1.2
70438384,0.6669533657,0.0206255841\h,2.1789915944,-1.2201018356,-0.257
6220681\c,-0.002638425,-2.9130281014,0.115144218\c,-1.2724872302,0.661
6600581,0.025896859\h,-2.1838589098,-1.2261690326,-0.2291005276\c,0.00
51839005,1.3744073148,0.2100075198\c,2.5720275595,1.4626188208,-0.0501
099402\o,-1.122010294,-3.4997952444,0.0975621477\o,1.1214571112,-3.477
6988408,0.2412006643\c,-2.5692631791,1.4589477392,-0.0439757055\h,0.00
362578,2.4338208133,0.4136863696\o,2.4560609679,2.7137739986,-0.056089
7043\o,3.6484099028,0.8195399738,-0.0992630121\o,-2.4479168061,2.71002
61859,-0.0674872731\o,-3.649476791,0.8204293369,-0.0759410224\\version
=es64l-g16revc.01\hf=-796.4411607\s2=2.009606\s2-1=0.\s2a=2.000057\rms
d=7.974e-09\rmsf=1.010e-05\dipole=-0.0117947,0.0710095,-0.0585161\quad
rupole=-29.2116018,-28.1660839,57.3776857,-0.1085821,-0.403776,4.37871
17\pg=c01 [x(c9h3o6)]\\@
```

Listing S6. Optimized Cartesian coordinates of the ligand in the analyte ethanol.

```
1\1\ginc-bdw06\fopt\uwb97xd\6-311++g(d,p)\c9h3o6(3-,3)\++++++++\08-mar
-2025\0\\#p wb97xd/6-311++g(d,p) opt=tight guess=read geom=check scrf=
(solvent=ethanol) int=ultrafine nosymm freq=noraman pop=full\\1,3,5-bt
c\\-3,3\c,0.0528616358,-1.4345288406,0.0129357698\c,1.2438919462,-0.71
61099373,0.0101285346\c,-1.250272876,-0.6661749525,0.0060848893\c,1.26
9885786,0.6670839676,0.0025846119\h,2.1809487151,-1.2592875603,0.00853
60965\c,0.0356939509,-2.9348182854,-0.0028394069\c,-1.2532843289,0.717
8495133,0.006623708\h,-2.1748685699,-1.2236020528,0.0028326255\c,-0.04
7085519,1.4106745208,0.0114533636\c,2.5596125629,1.4382563308,-0.02250
68934\o,-1.0964637262,-3.4822606315,-0.108356405\o,1.1387938897,-3.537
7770885,0.0864568779\c,-2.5820240196,1.4911128196,0.009264311\h,-0.028
9825522,2.4896648122,0.0230091642\o,2.457221805,2.6951920711,-0.061057
7354\o,3.6386532626,0.7879475759,-0.0053240879\o,-2.5072045593,2.74287
80648,0.0415879491\o,-3.6373774031,0.8138996728,-0.0214133727\\version
```

```
=es64l-g16revc.01\hf=-796.4262394\s2=2.067551\s2-1=0.\s2a=2.002048\rms
d=8.892e-09\rmsf=4.199e-06\dipole=-0.0364965,0.0145744,0.0624407\quadr
upole=-29.4598351,-28.3458745,57.8057096,1.0333799,-0.3108664,0.007992
3\pg=c01 [x(c9h3o6)]\\@
```

Listing S7. Optimized Cartesian coordinates of the ligand in the analyte toluene.

```
1\1\ginc-bdw06\fopt\uwb97xd\6-311++g(d,p)\c9h3o6(3-,3)\++++++++\08-mar
-2025\0\\#p wb97xd/6-311++g(d,p) opt=tight guess=read geom=check scrf=
(solvent=toluene) int=ultrafine nosymm freq=noraman pop=full\\1,3,5-bt
c\\-3,3\c,0.0536313546,-1.4370908192,0.0175635561\c,1.2434661418,-0.71
97149189,0.0384840391\c,-1.2520534986,-0.6683704853,0.0486820672\c,1.2
741368803,0.6697199285,0.012345002\h,2.1811520026,-1.2641676125,0.0515
931732\c,0.0350272698,-2.9406061339,-0.079674448\c,-1.2538923917,0.722
0649606,0.008435543\h,-2.180531849,-1.2206684844,0.1092626568\c,-0.048
6429794,1.4099193648,-0.026161201\c,2.568860567,1.4442965583,0.0137633
114\o,-1.0513457645,-3.4576107361,-0.4604195409\o,1.0900096112,-3.5664
099912,0.2060309871\c,-2.5884377313,1.4925911158,0.0271976566\h,-0.025
7671741,2.4901343713,-0.0619817125\o,2.4627860408,2.7028011322,-0.0033
821895\o,3.6498059503,0.795464365,0.0326801392\o,-2.5684552421,2.67270
44164,-0.397979589\o,-3.5897491877,0.8749429685,0.4635605491\\version=
es64l-g16revc.01\hf=-796.2191591\s2=2.068162\s2-1=0.\s2a=2.002099\rmsd
=5.799e-09\rmsf=6.187e-05\dipole=-0.06159,-0.0726963,0.1253873\quadrup
ole=-28.6938369,-26.9630494,55.6568863,1.4696865,-0.7119429,0.0952358\
pg=c01 [x(c9h3o6)]\\@
```

Listing S8. Optimized Cartesian coordinates of the ligand in the analyte acetone.

```
1\1\ginc-bdw06\fopt\uwb97xd\6-311++g(d,p)\c9h3o6(3-,3)\++++++++\08-mar
-2025\0\\#p wb97xd/6-311++g(d,p) opt=tight guess=read geom=check scrf=
(solvent=acetone) int=ultrafine nosymm freq=noraman pop=full\\1,3,5-bt
c\\-3,3\c,0.052561391,-1.4347429936,0.0135644243\c,1.2440133556,-0.716
1002463,0.0114772918\c,-1.2506651276,-0.6663005051,0.0074437098\c,1.27
0113452,0.6667684538,0.0028776974\h,2.1809628882,-1.2595476382,0.01183
17062\c,0.0354720505,-2.9350795921,-0.0023726685\c,-1.2536377106,0.717
4173072,0.0039827712\h,-2.1752479488,-1.2238325999,0.0070057103\c,-0.0
469644375,1.4102188315,0.0061958901\c,2.5600608074,1.4384250017,-0.016
6208534\o,-1.096539034,-3.4826369767,-0.1091260202\o,1.1385477899,-3.5
379481283,0.0880440717\c,-2.5820931402,1.4916074087,0.0045748137\h,-0.
0290531197,2.4892237735,0.011507591\o,2.4574766979,2.6955546983,-0.048
1331896\o,3.6391409104,0.7881644343,-0.001752461\o,-2.5058303786,2.743
6822652,0.0154446638\o,-3.638318446,0.8151265062,-0.0059451483\\versio
n=es64l-g16revc.01\hf=-796.4215507\s2=2.067484\s2-1=0.\s2a=2.002044\rm
sd=8.704e-09\rmsf=3.654e-06\dipole=-0.0366269,0.0133038,0.0559571\quad
rupole=-29.4551091,-28.3557601,57.8108692,1.0302268,-0.4087813,0.06786
15\pg=c01 [x(c9h3o6)]\\@
```

Listing S9. Optimized Cartesian coordinates of the ligand in the analyte 1,3,5-TMB (mesitylene).

```
1\1\ginc-bdw05\fopt\uwb97xd\6-311++g(d,p)\c9h3o6(3-,3)\++++++++\08-mar
-2025\0\\#p wb97xd/6-311++g(d,p) opt=tight guess=read geom=check scrf=
(solvent = mesitylene ) int =ultrafine nosymm freq=noraman pop=full\\1
,3,5-btc\\-3,3\c,-0.0000085437,-1.4264316972,0.0000585724\c,1.22803239
46,-0.6838159442,-0.0037141865\c,-1.2280528666,-0.6838159244,0.0038987
505\c,1.2290010748,0.6995014123,0.0002543893\h,2.1756523686,-1.2133984
299,0.0011922004\c,0.0000200545,-2.825203315,-0.0001272617\c,-1.229014
8258,0.6995019289,-0.0002349788\h,-2.1756776062,-1.2133918799,-0.00097
24951\c,-0.0000066399,1.3959765645,-0.0000525312\c,2.5596402176,1.4758
669183,0.0064973233\o,-1.0318644111,-3.6315632446,0.0635966706\o,1.031
9525495,-3.6314916824,-0.0639645469\c,-2.5596500413,1.4758749605,-0.00
64555309\h,-0.0000069783,2.4791095218,-0.0000963805\o,2.480798026,2.72
```

98808824,0.005490751\o,3.6293290087,0.8117468008,0.0135053473\o,-2.480
7970237,2.7298887227,-0.0056217971\o,-3.6293467577,0.8117644055,-0.013
2542959\\version=es64l-g16revc.01\hf=-796.219843\s2=2.045341\s2-1=0.\s
2a=2.001106\rmsd=8.761e-09\rmsf=3.538e-05\dipole=-0.0000212,-1.8783633
,0.0000232\quadrupole=-31.3938298,-21.7406904,53.1345201,0.0005108,-0.
035208,-0.001251\pg=c01 [x(c9h3o6)]\\@

Table S1. Quasiharmonic DFT thermodynamic properties.

Energy component (in au)	Solvent				
	Acetonitrile	N,N′-dimethylformamide	Chloroform	Methanol	Water
Zero-point correction	0.100479	0.100006	0.102119	0.100015	0.100533
Enthalpy correction	0.115099	0.114480	0.115839	0.114486	0.115141
Free Energy correction	0.056138	0.055844	0.060692	0.055840	0.056190
Quasiharmonic Free Energy correction	0.059122	0.058875	0.061692	0.058889	0.059221
SCF Energy	-796.432718	-796.433573	-796.332613	-796.431489	-796.441161
SCF Energy + ZPVE	-796.332239	-796.333567	-796.230494	-796.331474	-796.340628
Enthalpy	-796.317619	-796.319093	-796.216774	-796.317003	-796.326020
Free Energy	-796.376580	-796.377729	-796.271921	-796.375649	-796.384971
Free Energy + quasiharmonic correction	-796.373596	-796.374698	-796.270921	-796.372600	-796.381940
Correction (in kcal/mol)	1.87	1.90	0.63	1.91	1.90
Low frequencies before projection	34.7354	29.4630	55.2136	29.2399	37.3911
Low frequencies after projection	34.0346	26.4011	48.0573	25.4719	36.8

… Completion of Table S1.

Energy component (in au)	Solvent			
	Ethanol	Toluene	Acetone	1,3,5-TMBe
Zero-point correction	0.100176	0.103843	0.100226	0.104382
Enthalpy correction	0.114579	0.116790	0.114617	0.117665
Free Energy correction	0.056113	0.064468	0.056137	0.063290
Quasiharmonic Free Energy correction	0.059033	0.064557	0.059098	0.064389
SCF Energy	-796.426239	-796.219159	-796.421551	-796.219843
SCF Energy + ZPVE	-796.326063	-796.115316	-796.321325	-796.115461
Enthalpy	-796.311660	-796.102369	-796.306934	-796.102178
Free Energy	-796.370126	-796.154691	-796.365414	-796.156553
Free Energy + quasiharmonic correction	-796.367207	-796.154602	-796.362453	-796.155454
Correction (in kcal/mol)	1.83	0.06	1.86	0.69
Low frequencies before projection	24.3520	90.4130	26.8310	39.1057
Low frequencies after projection	16.8391	89.9988	16.662	31.3516

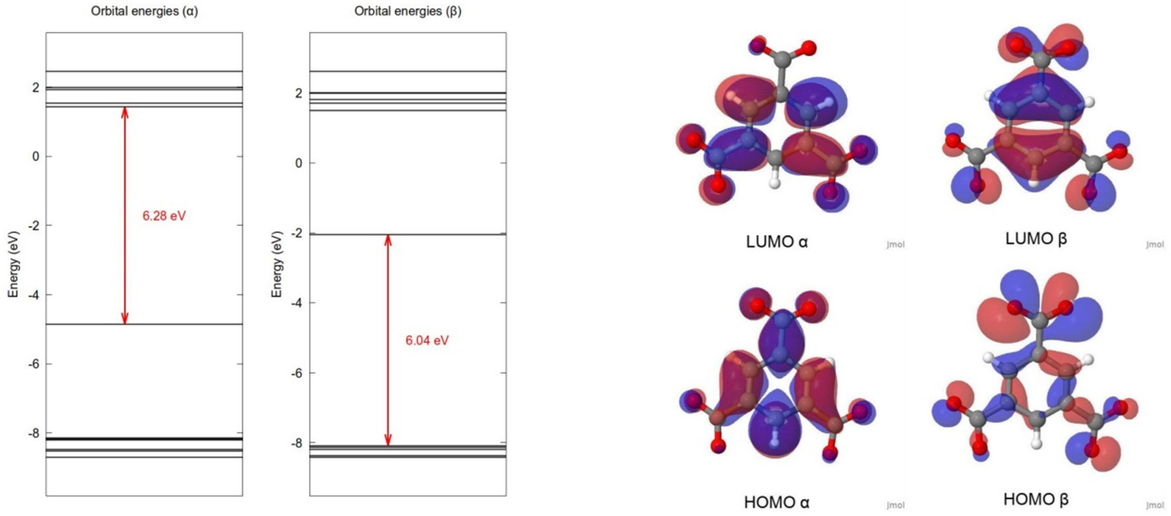

Figure S1. Energy diagram (left) and graphical representation of the ligand frontier orbitals (right) in the analyte acetonitrile. The homo-lumo energy gap is shown by a red arrow.

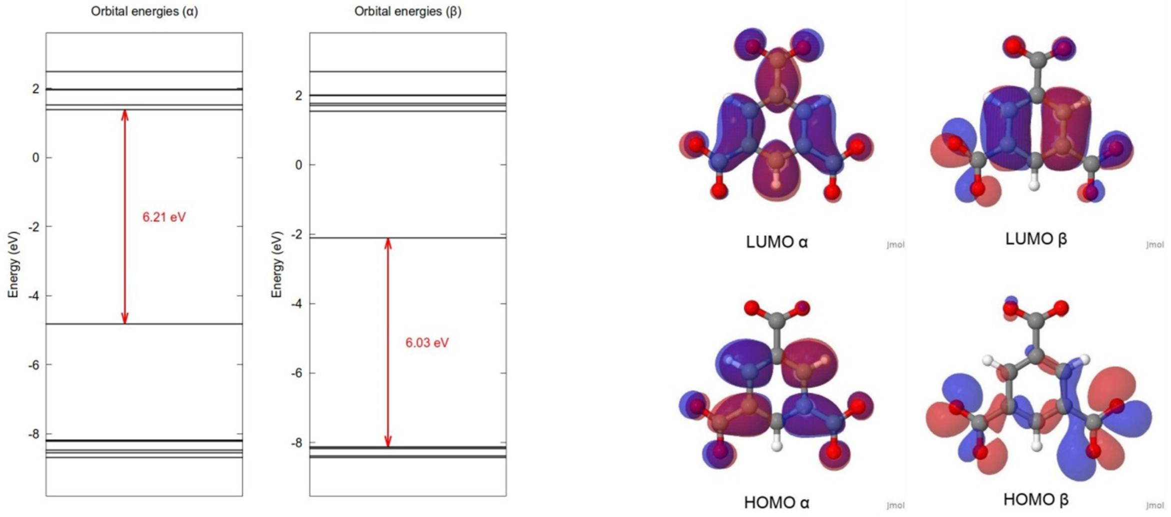

Figure S2. Energy diagram (left) and graphical representation of the ligand frontier orbitals (right) in the analyte n,n-dimethylformamide. The homo-lumo energy gap is shown by a red arrow.

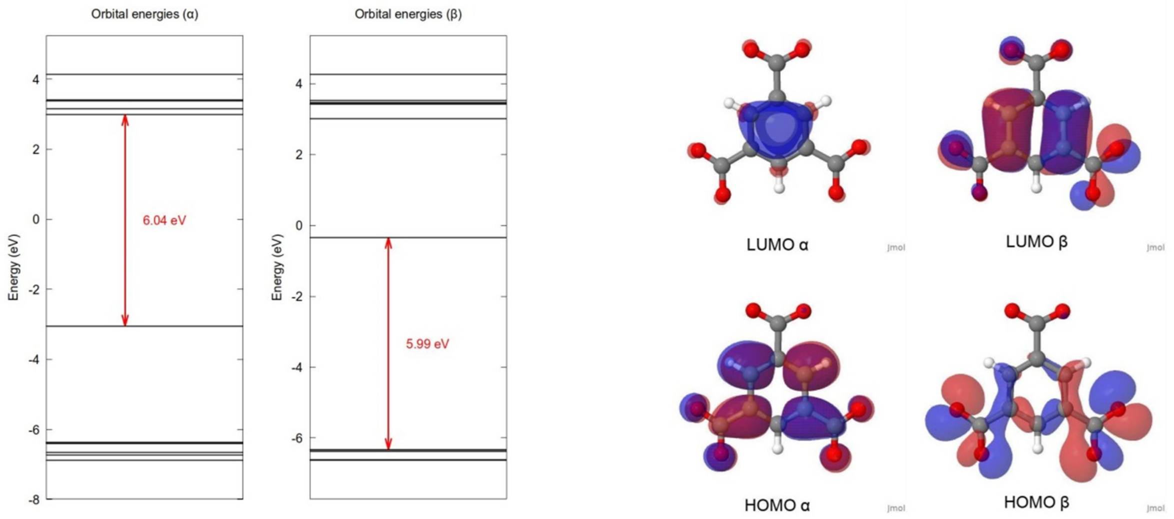

Figure S3. Energy diagram (left) and graphical representation of the ligand frontier orbitals (right) in the analyte chloroform. The homo-lumo energy gap is shown by a red arrow.

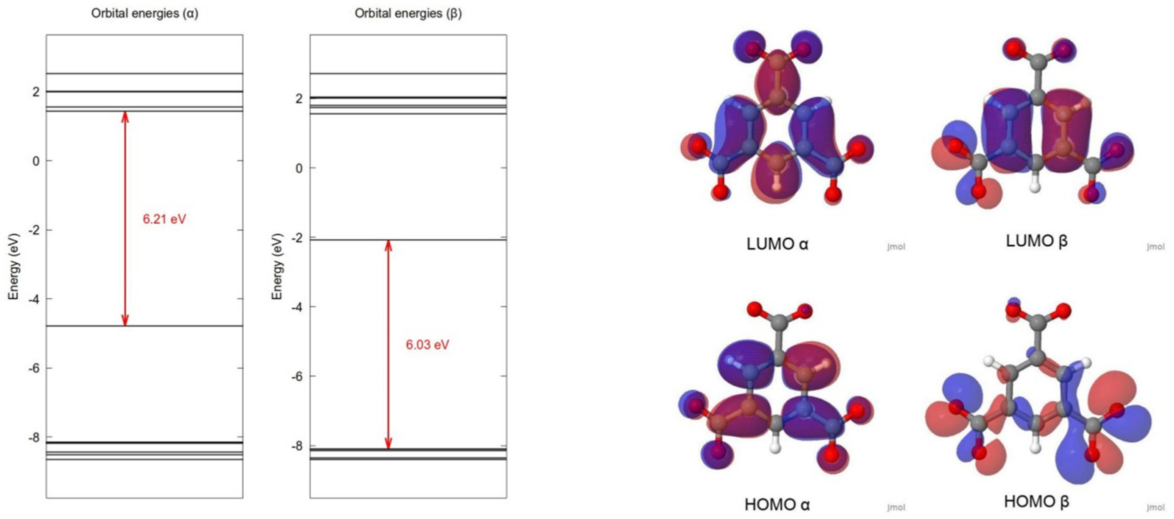

Figure S4. Energy diagram (left) and graphical representation of the ligand frontier orbitals (right) in the analyte methanol. The homo-lumo energy gap is shown by a red arrow.

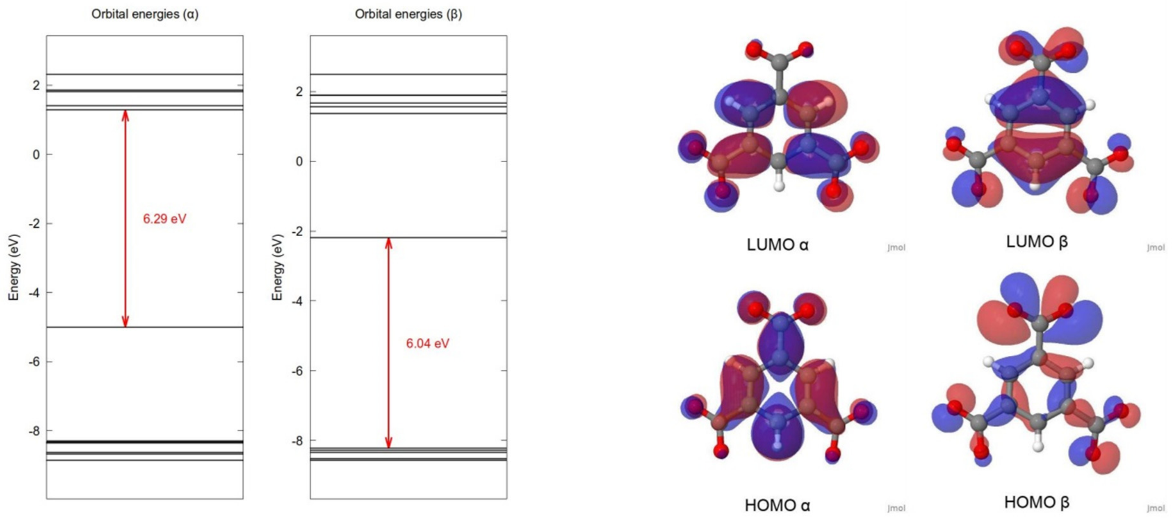

Figure S5. Energy diagram (left) and graphical representation of the ligand frontier orbitals (right) in the analyte water. The homo-lumo energy gap is shown by a red arrow.

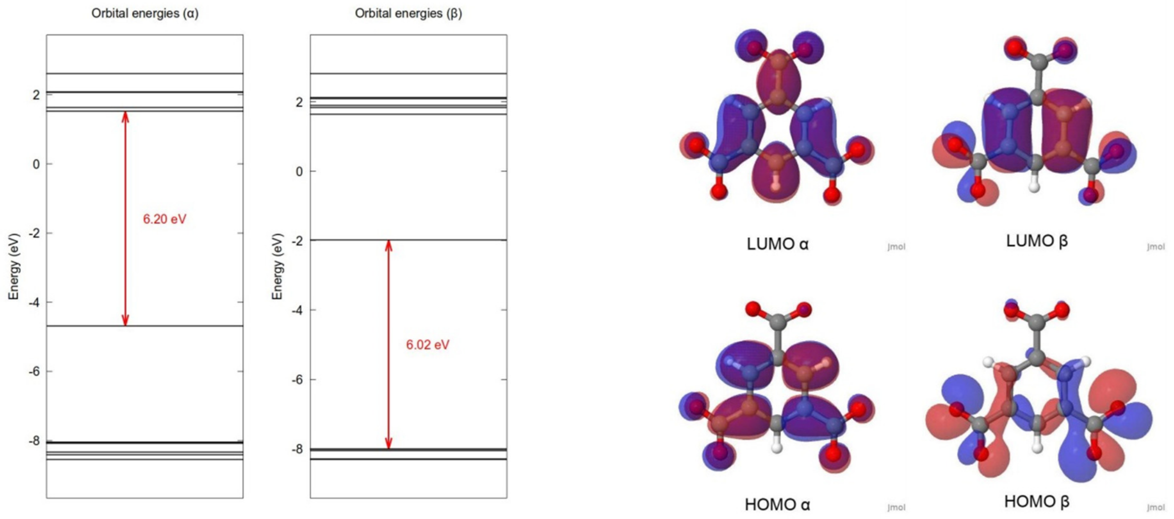

Figure S6. Energy diagram (left) and graphical representation of the ligand frontier orbitals (right) in the analyte ethanol. The homo-lumo energy gap is shown by a red arrow.

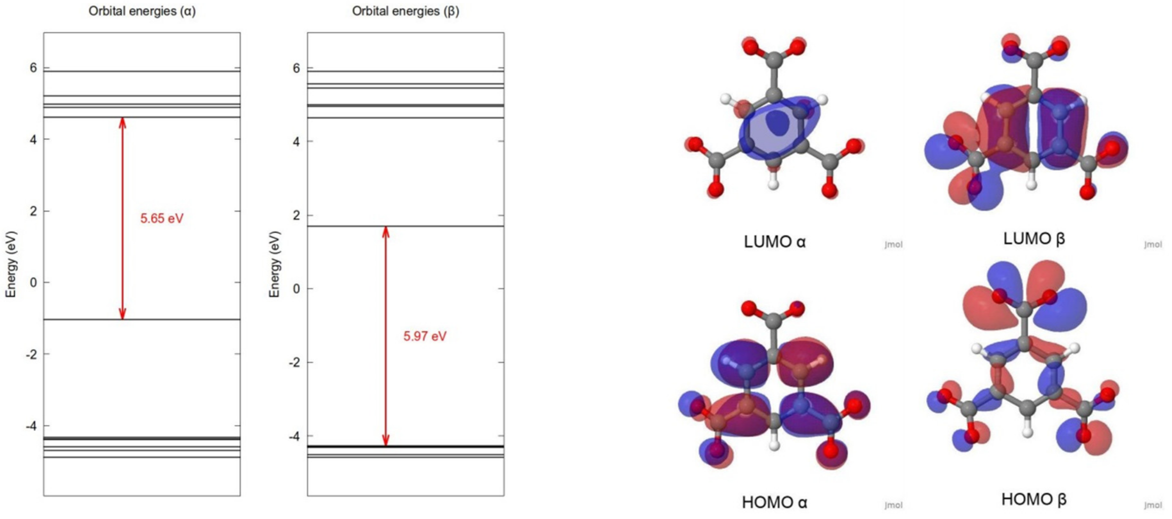

Figure S7. Energy diagram (left) and graphical representation of the ligand frontier orbitals (right) in the analyte toluene. The homo-lumo energy gap is shown by a red arrow.

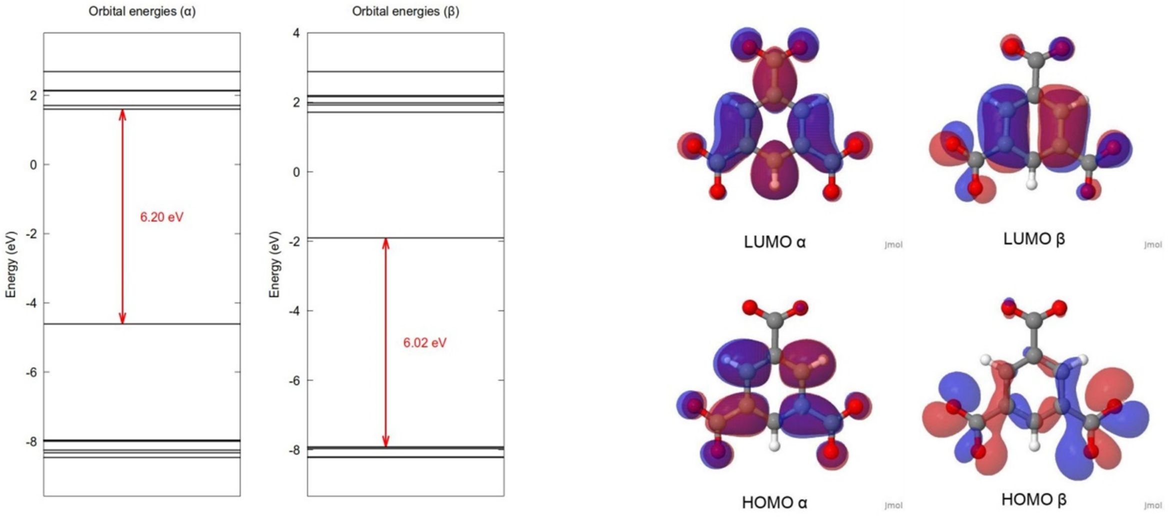

Figure S8. Energy diagram (left) and graphical representation of the ligand frontier orbitals (right) in the analyte acetone. The homo-lumo energy gap is shown by a red arrow.

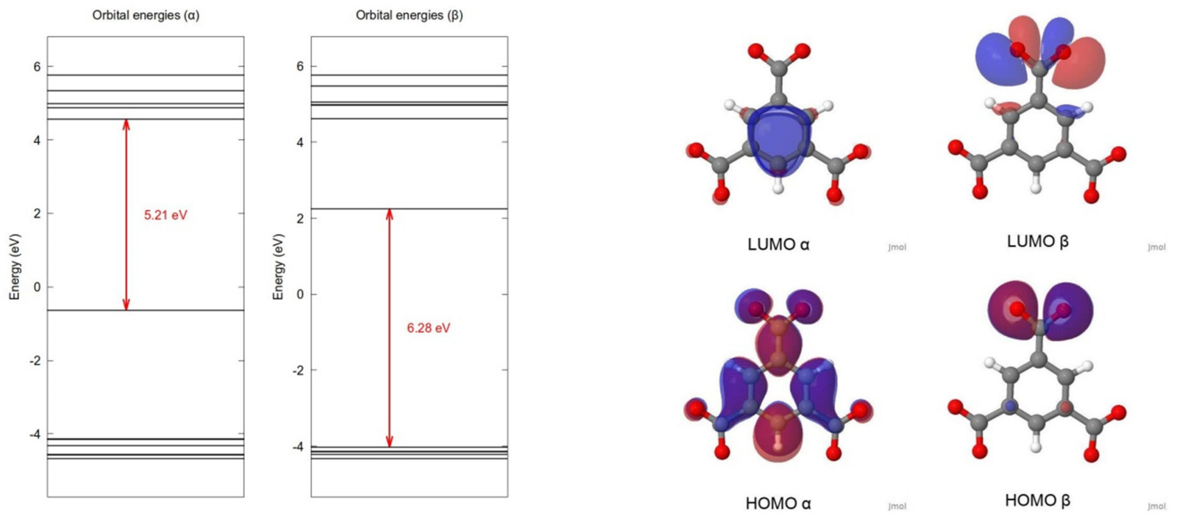

Figure S9. Energy diagram (left) and graphical representation of the ligand frontier orbitals (right) in the analyte mesitylene. The homo-lumo energy gap is shown by a red arrow.

Table S2. Computed absorption energies, excitation state character and transition weight.

Analyte	TDA (eV)	Excited state character	Transition weight (%)
Acetonitrile	6.3145	HOMO-3α → LUMOα	3.22
		HOMOα → LUMOα	42.21
		HOMO-3β → LUMOβ	3.22
		HOMOβ → LUMOβ	42.21
N,N′-dimethylformamide	6.2789	HOMO-2α → LUMOα	1.78
		HOMOα → LUMOα	42.72
		HOMO-2β → LUMOβ	1.78
		HOMOβ → LUMOβ	42.72
Chloroform	6.0456	HOMOα → LUMOα	42.96
		HOMOβ → LUMOβ	42.96
Methanol	6.1923	HOMO-2α → LUMOα	1.74
		HOMOα → LUMOα	42.74
		HOMO-2β → LUMOβ	1.74
		HOMOβ → LUMOβ	42.74
Water	6.3245	HOMO-3α → LUMOα	3.21
		HOMOα → LUMOα	42.34
		HOMO-3β → LUMOβ	3.21
		HOMOβ → LUMOβ	42.34
Ethanol	6.1628	HOMO-1α → LUMOα	1.11
		HOMOα → LUMOα	43.28
		HOMO-1β → LUMOβ	1.11
		HOMOβ → LUMOβ	43.28
Toluene	5.6123	HOMO-5α → LUMOα	2.17
		HOMO-3α → LUMOα	3.94
		HOMOα → LUMOα	37.94
		HOMO-5β → LUMOβ	2.17
		HOMO-3β → LUMOβ	3.94
		HOMOβ → LUMOβ	37.94
Acetone	6.1357	HOMOα → LUMOα	43.31
		HOMOβ → LUMOβ	43.31
1,3,5-TMB	5.2874	HOMOα → LUMOα	42.04
		HOMOα → LUMO+3α	4.66
		HOMOβ → LUMOβ	42.04
		HOMOβ → LUMO+3β	4.66

Experimental section

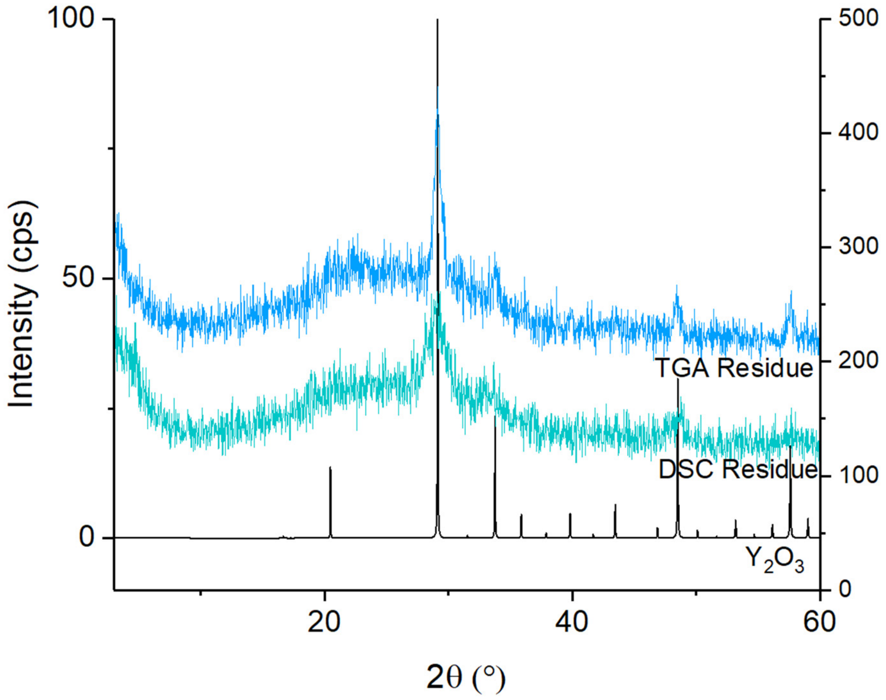

Figure S10. PXRD patterns of thermal analysis residue compared with the Y_2O_3 pattern.

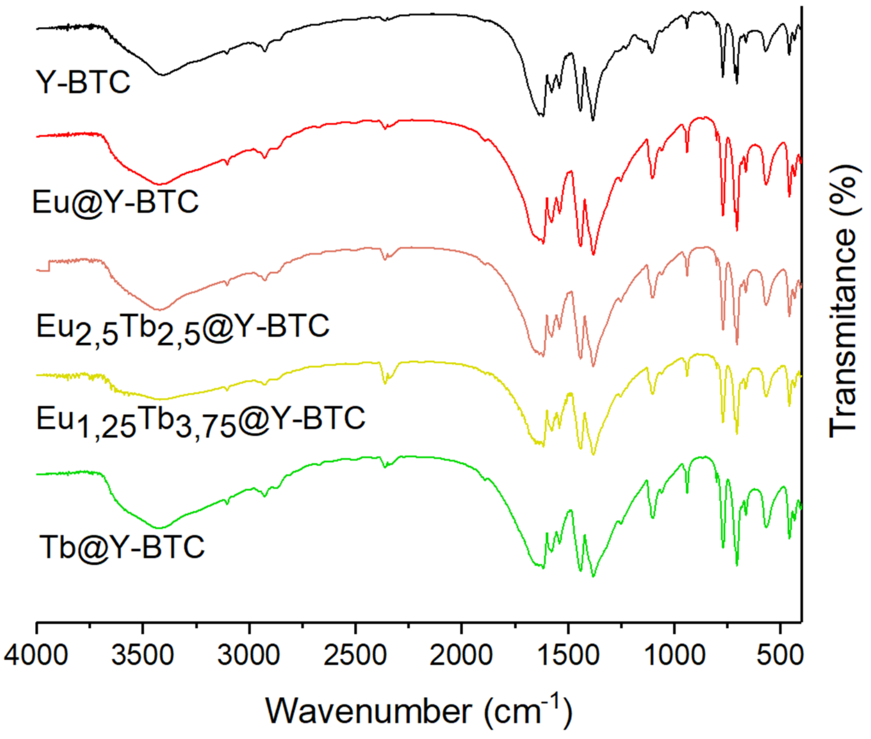

Figure S11. FTIR spectra of Y-BTC and Ln@Y-BTC.

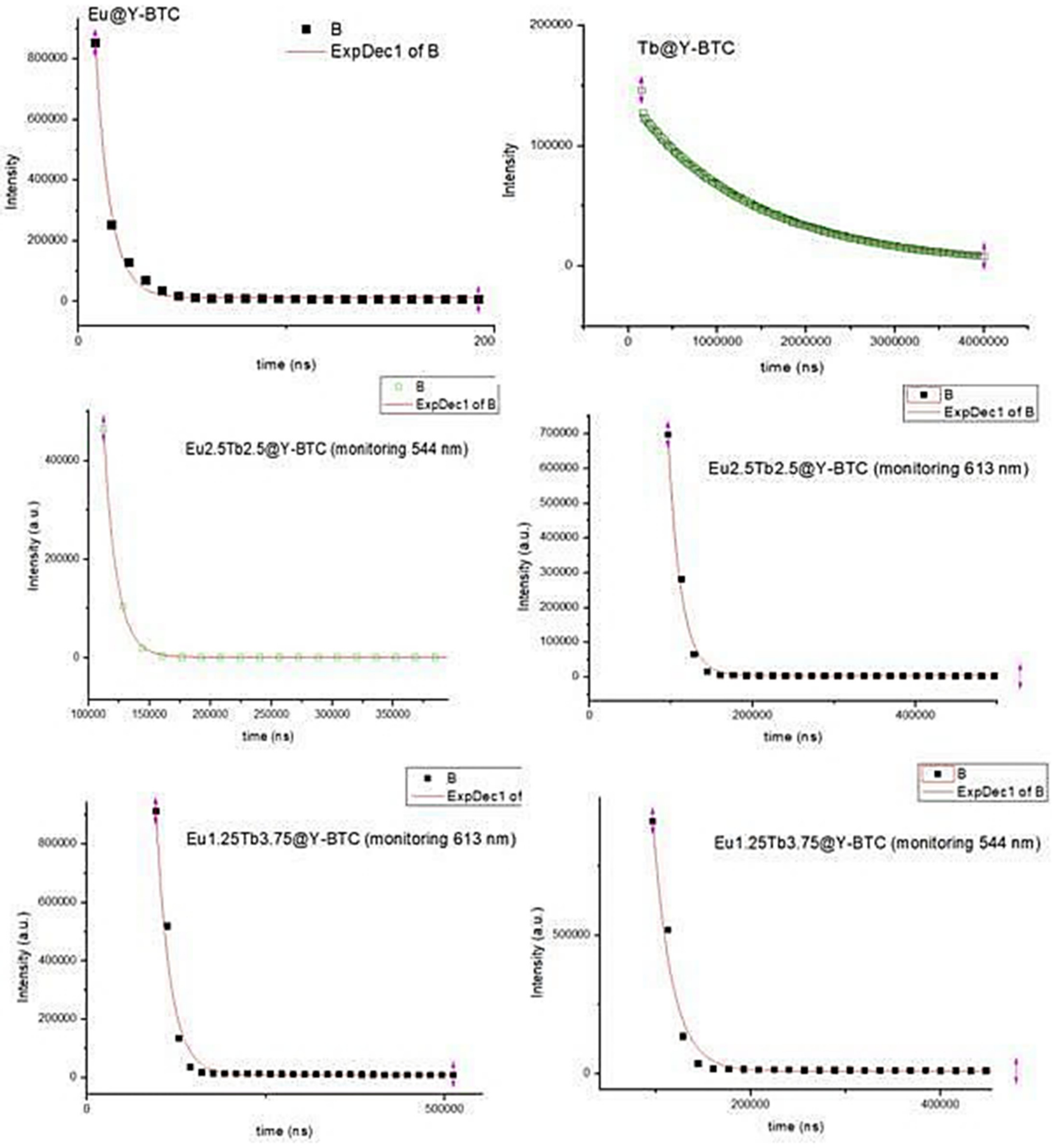

Figure S12. Decay profiles of the Ln@Y-BTC solid compounds.

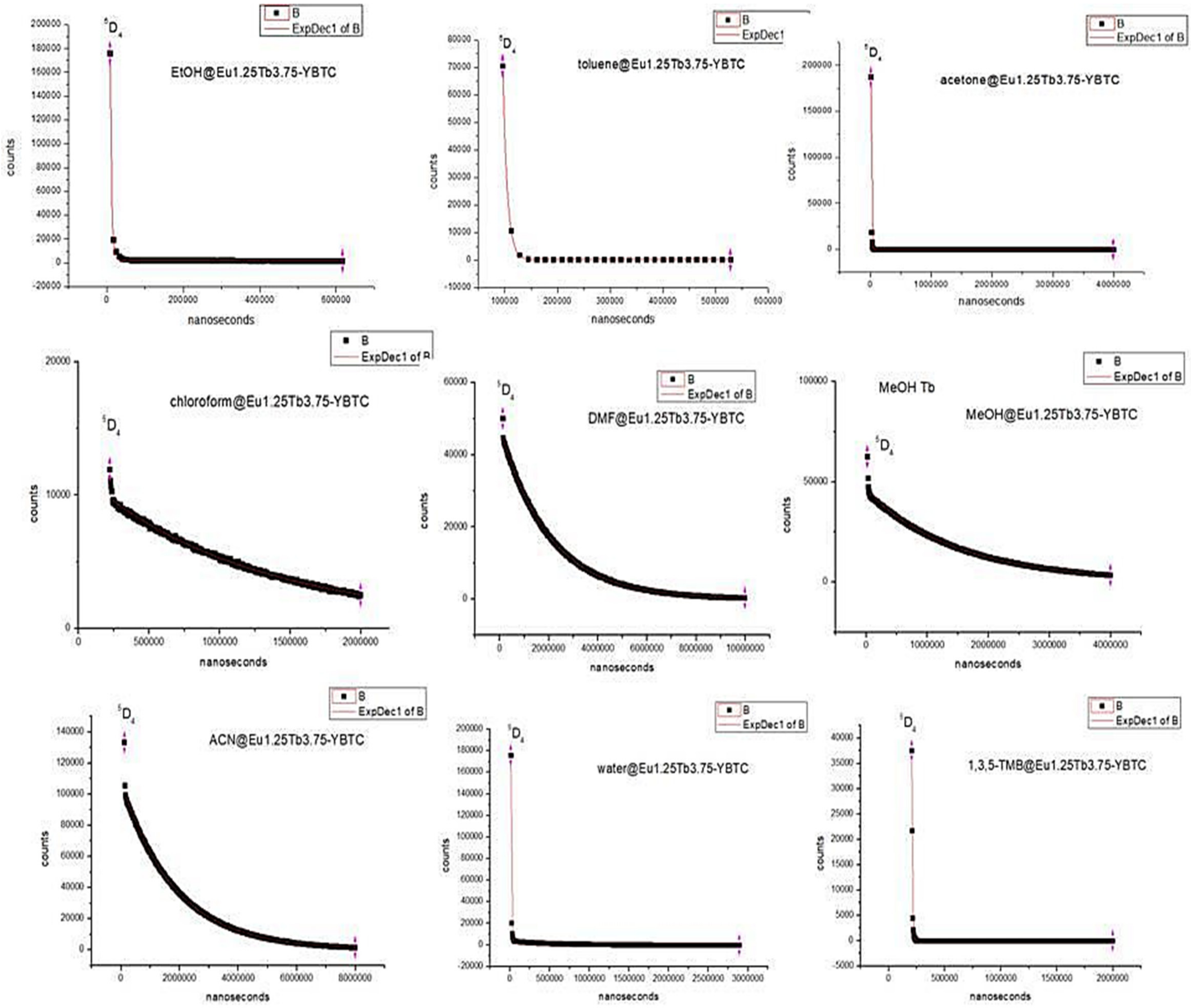

Figure S13. Terbium $^5D_4$ decay profiles of VOC@$Eu_{1.25}Tb_{3.75}$ suspensions.

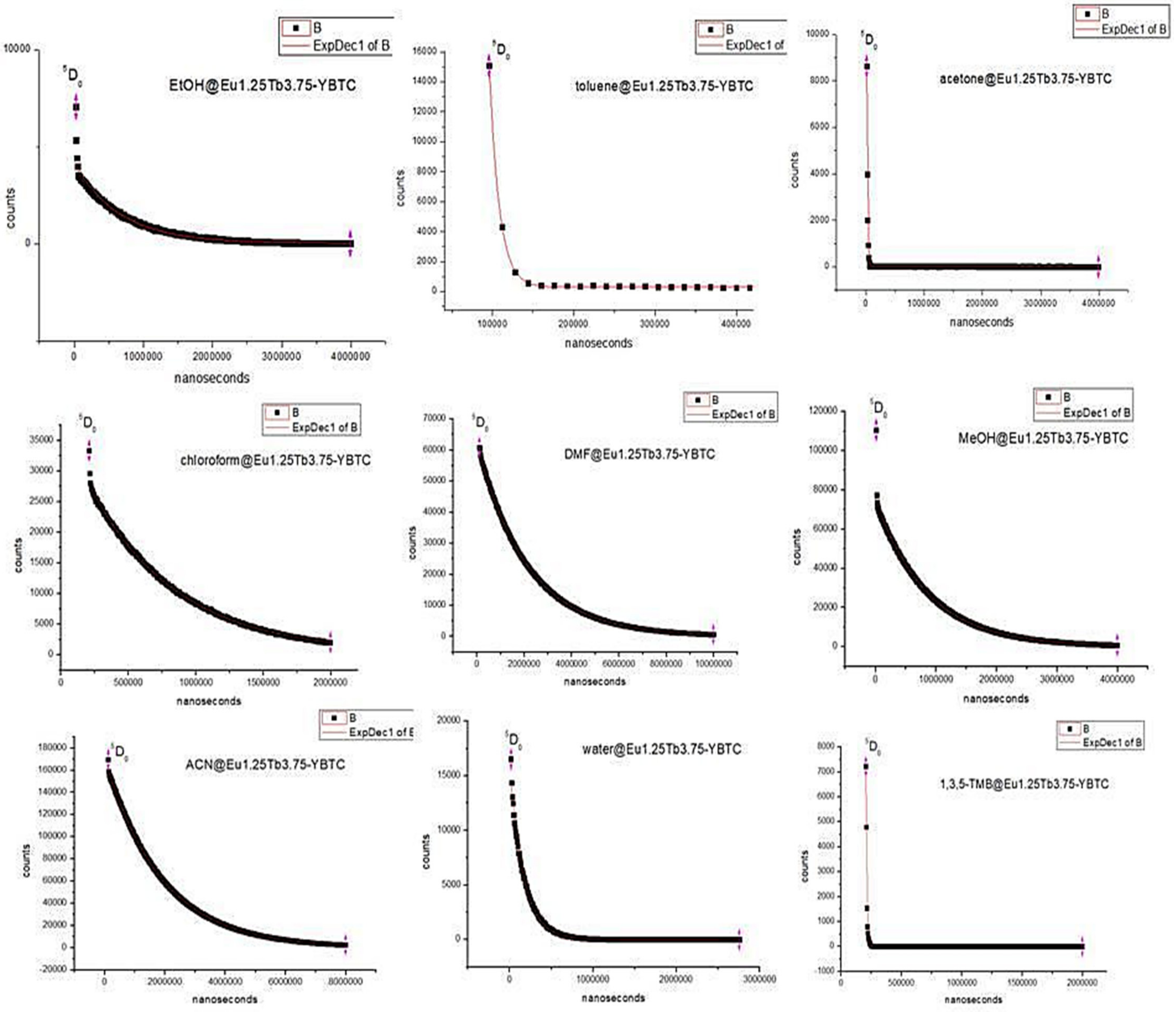

Figure S14. Europium $^5D_0$ decay profiles of VOC@$Eu_{1.25}Tb_{3.75}$ suspensions.

Table S3. Photophysical parameters of VOC@$Eu_{1.25}Tb_{3.75}$ suspensions.

VOC@$Eu_{1.25}Tb_{3.75}$	I_{tot}/I_{MD}	n	τ_{rad}/ms	k_{rad}/s^{-1}	k_{exp}/s^{-1}	k_{nrad}/s^{-1}	τ_{obs}/ms (Eu)	τ_{obs}/ms (Tb)	Q_{Eu}(%)
DMF@$Eu_{1.25}Tb_{3.75}$	6.618	1.428	3.54	282.34	473.93	191.58	2.11	2.00	59.6
ACN@$Eu_{1.25}Tb_{3.75}$	7.729	1.340	3.67	272.47	534.75	262.28	1.87	1.81	50.9
MeOH@$Eu_{1.25}Tb_{3.75}$	9.987	1.329	2.91	343.46	1196.17	852.7	0.836	1.46	28.7
EtOH@$Eu_{1.25}Tb_{3.75}$	12.344	1.361	2.19	455.92	1522.07	1066.14	0.657	0.0035	29.9
chlorofom@$Eu_{1.25}Tb_{3.75}$	8.152	1.443	2.78	358.84	1556.9	1198.05	0.6423	1.16	23.04
water@$Eu_{1.25}Tb_{3.75}$	9.83	1.33	2.95	338.82	6060.6	5721.77	0.165	0.0038	5.6
toluene@$Eu_{1.25}Tb_{3.75}$	41.192	1.496	0.49	2020.47	83333.3	81312.85	0.012	0.0008	2.42
acetone@$Eu_{1.25}Tb_{3.75}$	15.556	1.359	1.74	572.01	94339.62	93767.61	0.0106	0.00357	0.6
1,3,5-TMB@$Eu_{1.25}Tb_{3.75}$	35.563	1.499	0.56	1754.89	158730.16	156975.26	0.0063	0.0053	1.1

Table S4. Eu and Tb content in co-doped samples determined by ICP-AESP.

Sample	Lanthanide/%	
	Europium (Eu)	Terbium (Tb)
$Eu_{2.5}Tb_{2.5}$@Y-BTC	1.96±0.1	1.95±0.1
$Eu_{1.25}Tb_{3.75}$@Y-BTC	1.03±0.05	2.85±0.14
